# Composition of Immune Cells in Sporadic Vestibular Schwannomas with Different Tumor Volumes

**DOI:** 10.3390/cancers18030355

**Published:** 2026-01-23

**Authors:** Anna-Louisa Becker, Clara Helene Klause, Martin Sebastian Staege, Edith Willscher, Jonas Scheffler, Paola Schildhauer, Christian Ostalecki, Christian Strauss, Julian Prell, Christian Scheller, Stefan Rampp, Sandra Leisz

**Affiliations:** 1 Department of Neurosurgery, Medical Faculty, Martin Luther University Halle-Wittenberg, Ernst-Grube-Str. 40, 06120 Halle (Saale), Germany; 2 Department of Surgical and Conservative Pediatrics and Adolescent Medicine, Medical Faculty, Martin Luther University Halle-Wittenberg, Ernst-Grube-Str. 40, 06120 Halle (Saale), Germany; 3 Department of Internal Medicine IV, Oncology and Hematology, Medical Faculty, Martin Luther University Halle-Wittenberg, Ernst-Grube-Str. 40, 06120 Halle (Saale), Germany; 4 Department of Otorhinolaryngology, Head and Neck Surgery, Medical Faculty, Martin Luther University Halle Wittenberg, Ernst-Grube Str. 40, 06120 Halle (Saale), Germany; 5 Department of Dermatology, Medical Faculty, Friedrich Alexander University Erlangen-Nuremberg, Hartmannstraße 14, 91052 Erlangen, Germany; 6 Department of Neurosurgery, University Hospital Erlangen, Schwabachanlage 6, 91054 Erlangen, Germany; 7 Department of Neuroradiology, University Hospital Erlangen, Schwabachanlage 6, 91054 Erlangen, Germany

**Keywords:** vestibular schwannoma, immune cell infiltration, lymphocytes, macrophages, tumor microenvironment, multi-epitope ligand cartography, spatial analysis

## Abstract

Background/Objectives: Vestibular schwannoma (VS) is the most common benign tumor in the cerebellopontine angle. In preliminary studies, macrophage infiltration has been suggested to influence disease progression. However, the infiltration of other immune cells in VS remains largely unexplored. The aim of this study was to comprehensively characterize the immune cells in sporadic VS. Methods: Cryosections of five tumor samples from VS patients with different tumor volumes were examined. The abundance of fourteen immune-cell markers, one vascular marker, and two tumor markers were detected using multi-epitope ligand cartography (MELC). This enabled the spatial distribution and colocalization of immune- and tumor cell markers to be examined. Furthermore, using qPCR and bulk RNAseq, the mRNA levels of the immune-cell markers were examined in 204 VS samples of different tumor sizes. Results: VSs with greater tumor volumes showed an increased number of immune cells, more precisely T-helper cells (T_H_ cells), cytotoxic T cells (T_c_ cells), CD68^+^, and CD163^+^ macrophages, as well as CD279^+^ (PD-1) and CTLA4^+^ cells (*p* < 0.05). In addition, an increased number of CD274^+^ (PD-L1) tumor cells were detected in VSs with higher tumor volume (*p* < 0.05). Conclusions: These results indicate that an increased diversity of immune-cell subtypes influences VS tumor size. Thus, novel diagnostic and therapeutic options could be developed by targeting the tumor-associated immune-cell populations in VSs.

## Simple Summary

The size of sporadic vestibular schwannomas is known to be associated with a high number of macrophages. In many other tumors, macrophages, as well as other immune cells like monocytes or T cells, influence tumor size and tumor progression. Therefore, the aim of this study was to characterize the presence of different immune cells based on their surface proteins in sporadic vestibular schwannomas and to investigate their possible influence on tumor size. A better understanding of the immune cells present in VS could lead to the development of drug therapies for VS, which are not available for sporadic VS at the moment.

## 1. Introduction

The growth behavior of VSs is highly variable between patients, significantly influencing both therapeutic decisions and prognosis. While approximately 50% of patients show no signs of progression, some VSs exhibit a rapid increase in size [1]. Therapeutic strategies of VSs include monitoring tumor growth with magnetic resonance imaging (MRI) scans, radiosurgery, or microsurgical removal [2]. However, a limited understanding of mechanisms underlying tumor size progression contributes to the current lack of approved pharmacological treatments. This highlights a gap in therapeutic options. Increasing evidence in the literature indicates the relevance of tumor-associated macrophages (TAMs) within the tumor microenvironment in this process [3,4].

In malignant tumors, it is established that the polarization of macrophages towards an anti-inflammatory phenotype contributes to tumor progression and that other immune cells influence tumor progression [5]. Monocytes, pro-inflammatory macrophages, and several T-cell subtypes are particularly important [6]. Although certain immune-cell subtypes like cytotoxic lymphocytes (T_c_ cells) have been described to impact VS tumor volume, specific roles of other abundant cell types, such as immature or pro-inflammatory macrophages, T-helper cells (T_H_ cells), and regulatory T cells (T_reg_ cells) remain largely unexplored [4,7].

The presence of intra-tumoral immune cells and their activation status can be characterized by different surface molecules. CD45 (Leukocyte Common Antigen, LCA) is known as a general marker for all immune cells [8]. Immature macrophages or monocytes are identified by the markers CD14 and CD16, known as F_c_ gamma receptor III (FCGR3A) [9,10]. Pro-inflammatory (M1) macrophages are characterized by the expression of CD40 in addition to the expression of CD68 [11,12]. In contrast, anti-inflammatory and tumor growth-promoting (M2) macrophages express the surface molecules CD163 and CD68 [11].

T cells are characterized by the T-cell receptor CD3 as a surface marker [13]. They can be subdivided by the expression pattern of additional co-receptors. CD3^+^CD4^+^ T cells are referred to as T_H_ cells, CD3^+^CD8^+^ as T_c_ cells, and CD3^+^CD25^+^ as T_reg_ cells [14]. CD25 is also known as Interleukin-2 receptor alpha chain (IL2RA). The functional activities of these subsets are tightly regulated and influenced by various cell–cell interactions. CD279, better known as Programmed Cell Death Protein 1 (PD-1, gene name *PDCD1*), is located on the surface of T cells, while its ligand CD274 (Programmed Death-Ligand 1, PD-L1) is expressed on the surface of tumor cells, among others [15]. The interaction of CD279 with CD274 is involved in the prevention of apoptosis of tumor cells by T_c_ cells [15]. This mechanism has been described in many tumors and is now a target for checkpoint inhibitors in the treatment of malignant tumors. Cytotoxic T-lymphocyte-associated protein 4 (CTLA4) is a surface molecule frequently expressed on T_reg_ cells [16]. It competes with CD28 for binding to CD80, also known as B7.1, and CD86, also known as B7.2, on antigen-presenting cells. While costimulatory binding of CD80 and CD86 to CD28 promotes T-cell activation, binding of the tandem CD80 and CD86 to CTLA4 inhibits the activation of T cells [15].

Although macrophages are prominently represented, the composition, localization, and interactions of immune cells within the tumor microenvironment of VSs are still poorly understood. Therefore, the aim of this study was to analyze the occurrence and toponomy of immune-cell infiltration of sporadic VSs of different tumor volumes, as well as its quantification and correlation to tumor progression. Multi-epitope ligand cartography (MELC), RNA sequencing, and quantitative real-time polymerase chain reaction (qPCR) were used to identify the different intra-tumoral immune-cell populations [17].

## 2. Materials and Methods

### 2.1. Study Design

The Ethics Committee of the University of Halle-Wittenberg approved the study (approval number: 2020-122). The study was conducted in accordance with the Declaration of Helsinki between 2020 and 2025. The patient cohort included patients with sporadic vestibular schwannoma, excluding those with NF2-related schwannomatosis, prior radiotherapy or tumor recurrence. This particular group constituted the study’s population, encompassing 277 patients (Figure S1). Preoperatively, the tumor volume of the patients was determined by MRI images as previously described in Leisz et al. [3]. MRI images that had a slice thickness of less than 2.5 mm and were acquired no more than six months prior to surgery were included. Written informed consent was given from each patient prior to surgery. Preoperatively, the hearing function of the patients was evaluated according to the guidelines of the American Academy of Otolaryngology-Head and Neck Surgery (AAO-HNS), modified according to Rahne et al. [18], and an additional category DS (Surditas for deafness) was introduced. Tumor samples from five patients were used to identify the immune-cell markers in tumor tissue using MELC. RNA was extracted from the tumor samples of 208 patients and the RNA level of immune-cell markers was examined (Figure S1).

### 2.2. RNA Isolation of VS Tumor Samples

As described previously [3,19], RNA was isolated from tumor samples. Briefly, the tumor samples were disrupted using a tissue lyzer (Qiagen, Hilden, Germany) and the RNA was extracted using the Qiagen AllPrep DNA/RNA/Protein Mini Kit (Qiagen, Hilden, Germany) according to the manufacturer’s instructions.

### 2.3. Quantitative Real-Time PCR

Quantification of cell surface protein mRNA level was performed using qPCR [3]. Briefly, RNA was transcribed into cDNA using the RevertAid First Strand cDNA Synthesis kit (Thermo Fisher Scientific, Waltham, MA, USA). The cDNA used for qPCR originated from the tumors, of different sizes, of 204 patients (baseline data, Table S1). Specific forward and reverse primers were synthesized from Invitrogen (Thermo Fisher Scientific, Waltham, MA, USA). Sequences are shown in Table S2. For analysis, the 2^−ΔΔCT^ method was utilized [20]. Glycerin aldehyde 3-phosphate dehydrogenase (*GAPDH*) was used as a housekeeping gene.

### 2.4. RNAseq Analysis of VS Tumor Samples

The RNA used for the RNAseq analysis originated from 10 VSs (Figure S1) and was prepared as described in Section 2.2. RNAseq was performed as described previously [19]. Briefly, 1 µg of RNA was analyzed using the Illumina Novaseq6000 system (Novogene, Cambridge, UK). These data were further processed with the Galaxy Server (https://usegalaxy.eu/, accessed on 13 December 2023), as described in Wieland et al. [21]. The reads were mapped to the human genome hg38 with Hisat2 and quantified using *featureCounts* of the Subread package. Based on their size, the VS samples were grouped into small (tumor volume < 5 cm^3^) and large VSs (tumor volume > 5 cm^3^; Table S3) for further analysis.

### 2.5. Deconvolution Analysis

The different immune-cell populations were identified and enumerated with the digital cytometry tool CIBERSORTx (v.1.0), as previously described [22]. In brief, as the signature file, the leukocyte gene signature matrix LM22 [23] was used to analyze the cell composition of immune cells in our bulk RNAseq data of VS tumor samples with default settings on the CIBERSORTx online platform.

### 2.6. Multi-Epitope Ligand Cartography

The tumors examined in the MELC originate from five patients and had very different tumor volumes (Table S4). MELC was performed as described previously for melanoma [24,25,26]. Cryosections were used for the MELC (Supplemental method S1). As a control, S100B immunohistochemical staining (IHC) was performed on tissue used for MELC (Supplemental method S2, Table S5). At first, a specific antibody panel for MELC analysis of VS sections was established. CD56 was used as a tumor cell marker. Antibodies against CD14, CD16, CD40, CD68, and CD163 were used as markers for macrophages. Antibodies against CD3, CD4, CD8, and CD25 were used to analyze T cells. In addition, antibodies against CD31, CD274, CD279, and CTLA4 were examined, with CD31 being a marker for blood vessels. The cryosections used were prepared as described in Supplemental Method S1. Briefly, cryosections were fixed in −20 °C cold acetone. Subsequently, cryosections were rehydrated with phosphate-buffered saline (PBS; pH 7.4; Lonza, Verviers, Belgium), incubated with normal-goat serum, and washed with PBS. The cryosection, as well as the diluted antibodies (Table S6), were placed in MELC robotic technology. Robotically assisted fluorescence microscopy images of selected sections of the sample were obtained to determine the autofluorescence, followed by bleaching of the cryosection. All sections had a defined size of 900 × 900 µm. Robotically assisted, the cryosection was then stained with the different antibodies, respectively. Then, fluorescence microscopy images of the image sections were obtained, and the cryosection was bleached, washed with PBS, and stained again. The staining and bleaching cycles were repeated for each individual antibody stain.

### 2.7. Processing and Analysis of the Fluorescence Microscopy MELC Images

The images were stained and overlaid using CellProfiler software (version 4.2.4; Broad Institute, Cambridge, MA, USA) [27]. The *GrayToColor*, *MakeProjection*, and *SaveImage* modules were used for this purpose. To detect double-positive cells, the images were overlaid using ImageJ (version 1.53t) and the tool *ImageCalculator*. The *AND* function was used to obtain only signals present on both images. A quantification of the signals was also performed with the *IdentifyPrimaryObjects* function of the software CellProfiler (Figure S2).

### 2.8. Statistical Analysis

qPCR data were correlated with clinical parameters such as tumor volume, Koos grade (Table S7), hearing class, and age at surgery. Since the hearing classes are ordinally scaled according to the AAO-HNS classification and the data are not normally distributed, Spearman’s rank correlation was utilized. Analyses were calculated with R 4.0.5 [28]. The data from MELC and RNAseq were divided into small (tumor volume < 5 cm^3^) and large VSs (tumor volume > 5 cm^3^). The differences in the investigated immune-cell markers and tumor surface markers between the groups were calculated using the Mann–Whitney test in GraphPad Prism version 10.4.2 (GraphPad Software, Boston, MA, USA). The medians are shown as a horizontal line. The boxes range from the 25th to the 75th percentile, and the whiskers extend from the smallest to the largest value. Since this study has an explorative experimental design, it was not corrected for multiple testing.

## 3. Results

### 3.1. Characterization of VS Tissue Samples

For visualization of VS tumor cells in the MELC, VS tumor cell markers [29,30] CD56 and S100B were used (Figure S3). Since MELC analysis detects cell surface markers, the fluorescence signal for CD56 as a tumor marker was used (Figure S3a, Table S8). However, using IHC, the tissue was strongly positive for S100B (Figure S3b). The blood vessels were stained in the MELC sections using the marker CD31 (Figure S4).

### 3.2. Increased Level of the Lymphocytes in Large VS

**Figure 1:**
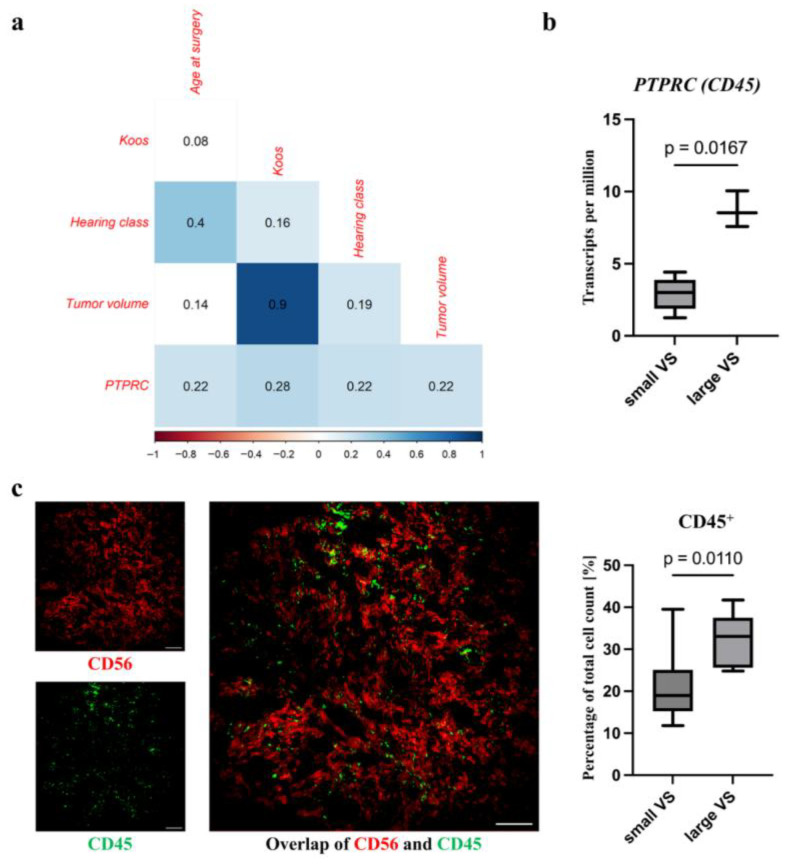
Analysis of the CD45^+^ lymphocytes in VS. Correlation analysis of the indicated marker mRNA level and clinical parameters in tumor samples of 204 VS patients using Spearman’s rank test. The correlation coefficient r is shown. Significant positive correlations (*p* < 0.05) are plotted in blue. Non-significant correlations are plotted uncolored (**a**). The statistical difference in PTPRC RNA levels in tumor samples of small and large VS detected using RNAseq was calculated by Mann–Whitney test (**b**). A representative staining of CD56 and CD45 in a MELC image of a VS is illustrated. Percentage of CD45^+^ cells of total cell count in large and small VSs, and the group difference analyzed by Mann–Whitney test is shown. Scale bar = 100 µm (**c**).

The correlation analysis of the qPCR data revealed a weak positive correlation of protein tyrosine phosphatase receptor type C (*PTPRC*, gene name of CD45), mRNA level with increased age at surgery (r = 0.22, *p* = 0.02; Figure 1a, Table S9), Koos grade (r = 0.28, *p* = 0.005), poor hearing (r = 0.22, *p* = 0.03), and tumor volume (r = 0.22, *p* = 0.04). Large VSs showed a higher *PTPRC* RNA level in the RNAseq analysis of VS tumor samples (Figure 1b; small VS, median transcripts per million (TPM): 2.99, IQR: 1.59; large VS, median TPM: 8.53, IQR: 1.24; *p* = 0.02; Table S10). Furthermore, the immune-cell marker CD45 was detectable in all VS sections (Figure 1c). Immune cells are shown to infiltrate the VS and are distributed in the tumor. However, there was a slightly higher density of cells in the proximity of some blood vessels. Large VS showed a higher number of CD45^+^ cells compared to small VS in the MELC analyses of VS tumor samples (Figure 1c; small VS, median 18.98%, IQR: 8.15; large VS, median: 33.06%, IQR: 6.91; *p* = 0.01, Table S11).

### 3.3. Macrophages Are More Abundant in VS with Higher Tumor Volume

#### 3.3.1. Increased mRNA Level of Monocyte and Different Macrophage Marker

**Figure 2:**
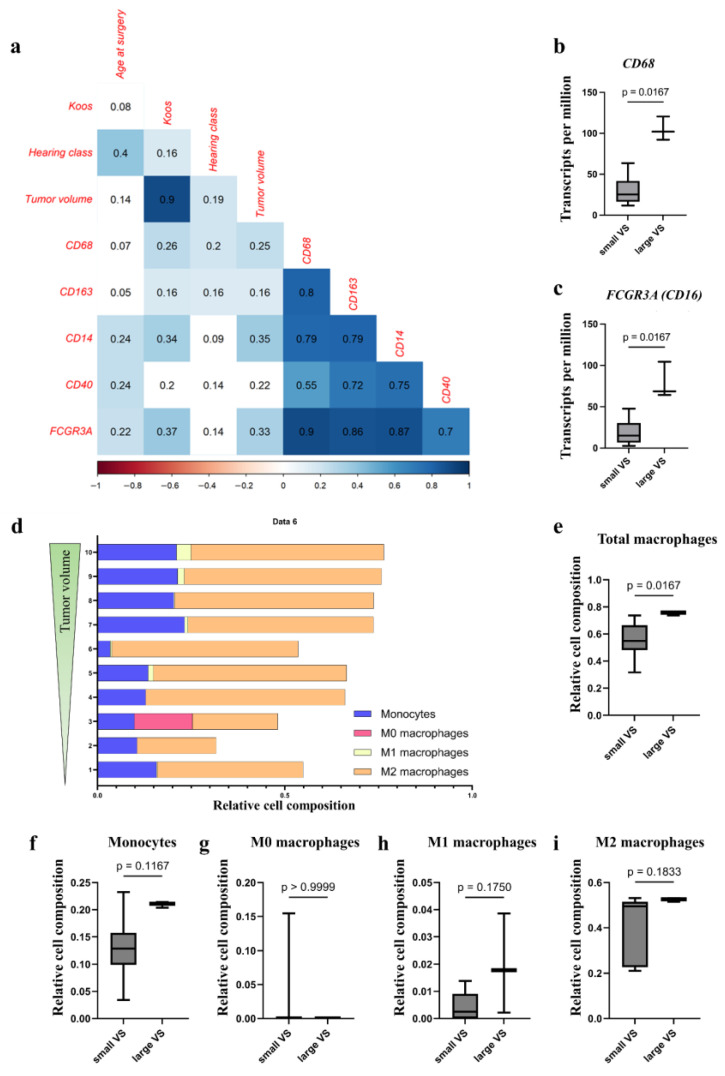
Macrophage marker mRNA level and macrophage amount in VSs with different tumor volumes. Correlation analysis of clinical data and *CD68*, *CD163*, *CD14*, *CD40*, and *FCGR3A* qPCR data in 204 VS tumor samples using Spearman’s rank test. The correlation coefficient r is shown. Significant positive correlations (*p* < 0.05) are plotted in blue. Non-significant correlations are plotted uncolored (**a**). Using bulk RNAseq, transcripts per million reads of *CD68* (**b**) and *FCGR3A* (**c**) in tumor samples from ten VS were calculated. Deconvolution analysis of macrophage distribution from RNAseq data is depicted. Shown are the relative cell compositions of monocytes, M0 macrophages, M1 macrophages, and M2 macrophages, whereas the tumors are sorted by size, with the smallest tumor assigned the number 1 and the largest tumor the number 10 (**d**). Differences in relative cell composition of small and large VSs in total number of macrophages (**e**), monocytes (**f**), M0 macrophages (**g**), M1 macrophages (**h**), and M2 macrophages (**i**) are shown. The differences in RNA level and immune-cell composition in small and large VSs were analyzed using the Mann–Whitney test.

The qPCR data showed a significant positive correlation of the macrophage marker levels of *CD68*, *CD163*, *CD14*, and *FCGR3A* (gene name of CD16) with Koos grade and tumor volume (r = 0.16 to 0.37, *p* < 0.05; Figure 2a, Table S8). The mRNA level of general macrophage marker *CD68* and M2 marker *CD163* also correlated positively with poor hearing, whereas monocyte marker *CD14* as well as M1 markers *CD40* and *FCGR3A* mRNA levels showed a positive correlation with the age at surgery (r = 0.22 to 0.24, *p* < 0.05). The macrophage marker levels correlated strongly with each other (Figure 2a).

Similar results were obtained in the transcriptome analysis of tumor samples of different sizes (Tables S12–S15). The mRNA level of *CD68* (small VS, median TPM: 25.27, IQR: 17.14; large VS, median TPM: 102.05, IQR: 14.23; *p* = 0.02; Figure 2b) and *FCGR3A* (small VS, median TPM: 15.22, IQR: 13.85; large VS, median TPM: 68.66, IQR: 20.10; *p* = 0.02; Figure 2c) of the large tumors were significantly higher than the mRNA level of the small tumors. In addition, the mRNA expression of *CD14* and *CD163* tended to be higher in large tumors than in small tumors (Figure S5).

Furthermore, the relative cell compositions of M0, M1, and M2 macrophages and monocytes in VSs of different sizes were calculated from the RNAseq data using deconvolution analysis (Figure 2d). The overall percentage of macrophages was significantly higher in large VSs (median: 0.76, IQR: 0.01) than in small VSs (median: 0.55, IQR: 0.15; Figure 2e, Table S16). The relative cell composition showed a tendency to be higher in large VSs (monocytes large VS, median: 0.21, IQR: 0.005; M1 macrophages large VS, median: 0.02, IQR: 0.02; M2 macrophages large VS, median: 0.53, IQR: 0.008) than in small VSs (monocytes small VS, median: 0.13, IQR: 0.04; M1 macrophages small VS, median: 0.002, IQR: 0.007; M2 macrophages small VS, median: 0.49, IQR: 0.20; Figure 2f, Tables S17–S19).

#### 3.3.2. Higher Level of TAM in Larger VSs

**Figure 3:**
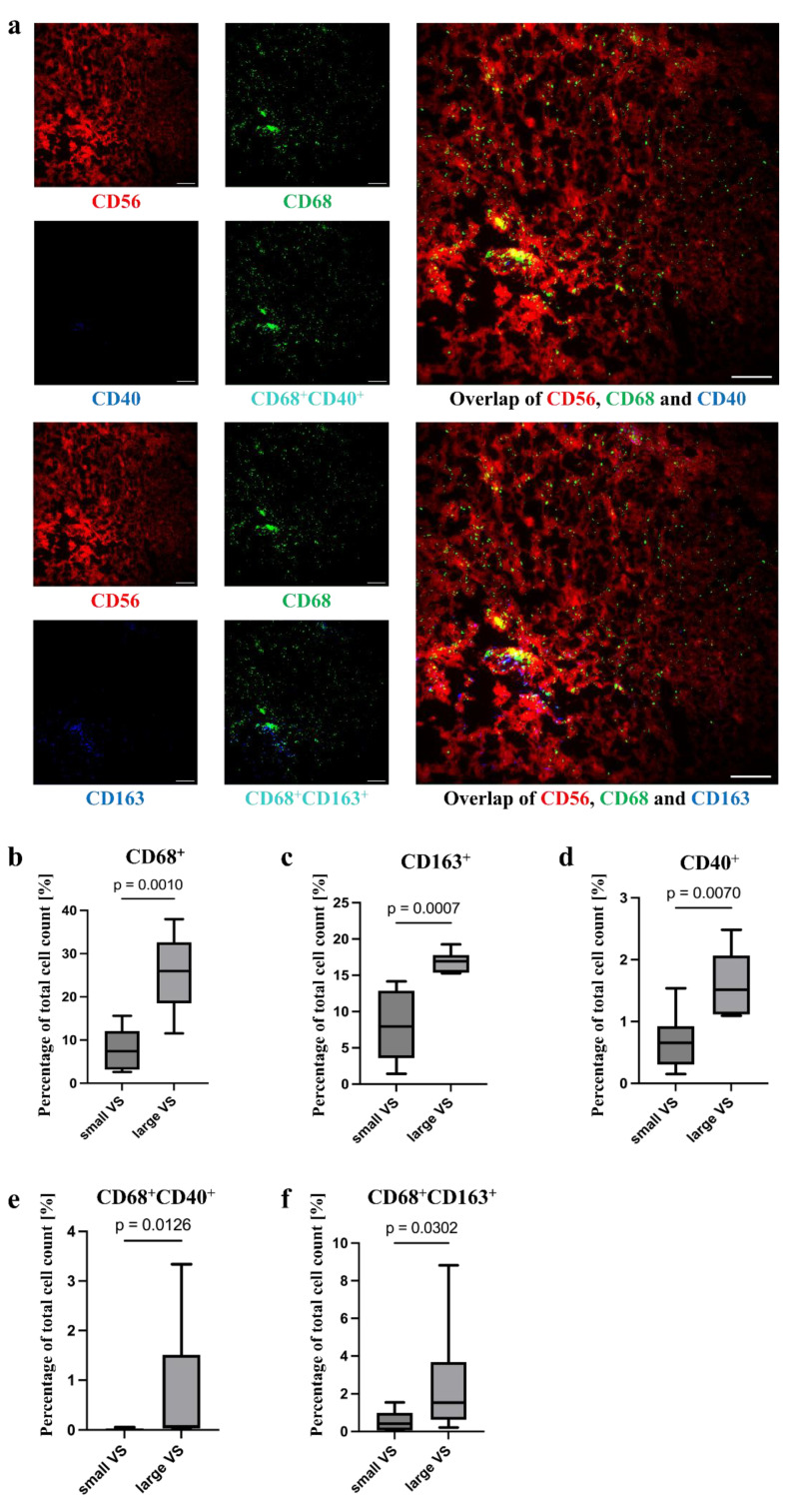
Distribution of TAM in VS. The CD68^+^, CD40^+^, and CD163^+^ cells in representative MELC images are shown. The MELC image section has a size of 900 × 900 µm, respectively. Fluorescence signals of CD56, CD68, CD40, and CD163 as well as CD68^+^CD40^+^ and CD68^+^CD163^+^ cells are exemplary visualized. Scale bar = 100 µm (**a**). In addition, the difference in the percentage of CD68^+^ (**b**), CD163^+^ (**c**), CD40^+^ (**d**), CD68^+^CD40^+^ (**e**), and CD68^+^CD163^+^ cells (**f**) to the total cell count in small and large VSs was calculated using the Mann–Whitney test (**b**–**f**). Analysis of CD68^+^CD14^+^ and CD68^+^CD16^+^ cells are shown in Figure S6.

The MELC analysis revealed CD68^+^ cells, which were visibly distributed in the tumor (Figure 3a, Table S20). Other macrophage markers were also detected in the tumor tissue (Tables S21 and S22). Some of these CD68^+^ macrophages were double positive for other macrophage-defining surface molecules. Thus, CD68^+^CD14^+^, CD68^+^CD16^+^, CD68^+^CD40^+^, and CD68^+^CD163^+^ macrophages could be identified, whereby there were differences in the number of cells positive for the surface molecules (Tables S23–S26, Figure 3a and Figure S6a). There was an accumulation of CD68^+^ cells around the blood vessels (Figure 3a). In some image sections, the CD14^+^ macrophages were also diffusely distributed in the tumor. In others, it appeared as if the CD14^+^ cells were mainly present in areas where fewer CD68^+^ cells were present. This was particularly evident in the larger tumors. CD16^+^ and CD163^+^ cells also appeared to be distributed within the tumor area. Many image sections showed an accumulation of CD16^+^ and CD163^+^ cells in areas where fewer or no CD14^+^ cells were present. However, in a smaller number of other sections, CD14^+^, CD16^+^, and CD163^+^ cells appeared together. It could be observed that the CD16^+^ cells mainly occur where the CD163^+^ cells were also present. The CD40^+^ cells were also distributed in the tumor. However, only a few cells could be seen that were diffusely distributed.

For CD68^+^ (median small VS 7.42%, IQR 7.01; median large VS 20.51%, IQR 17.85; *p* = 0.001; Figure 3b, Table S20), CD40^+^ (small VS, median: 0.66%, IQR: 0.53; large VS, median: 1.09%, IQR: 0.51; *p* = 0.01; Figure 3c, Table S21) and CD163^+^ cells (small VS, median: 7.94%, IQR: 7.65; large VS, median: 16.95%, IQR: 2.21; *p* < 0.001; Figure 3d, Table S22), as well as for CD68^+^CD40^+^ (small VS, median: 0%, IQR: 0.03; large VS: median: 0.07%, IQR 0.44; *p* = 0.01; Figure 3e, Table S23) and CD68^+^CD163^+^ (small VS, median: 0.42%, IQR: 0.73; large VS, median: 1.53%, IQR: 0.68; *p* = 0.03; Figure 3f, Table S24) cells, a significantly higher proportion of the total percentage of cells was detected in the large tumors. In addition, a tendency towards higher percentage of CD68^+^CD14^+^ (median: 1.16%, IQR: 1.35; Figure S6b, Table S25) and CD68^+^CD16^+^ cells (median: 0.39%, IQR: 0.73; Table S26) was detected.

### 3.4. Increased Amount of T Cells in Larger VSs

#### 3.4.1. Increased Level of T-Cell Surface Molecule Expression in VSs with Higher Tumor Volume

**Figure 4:**
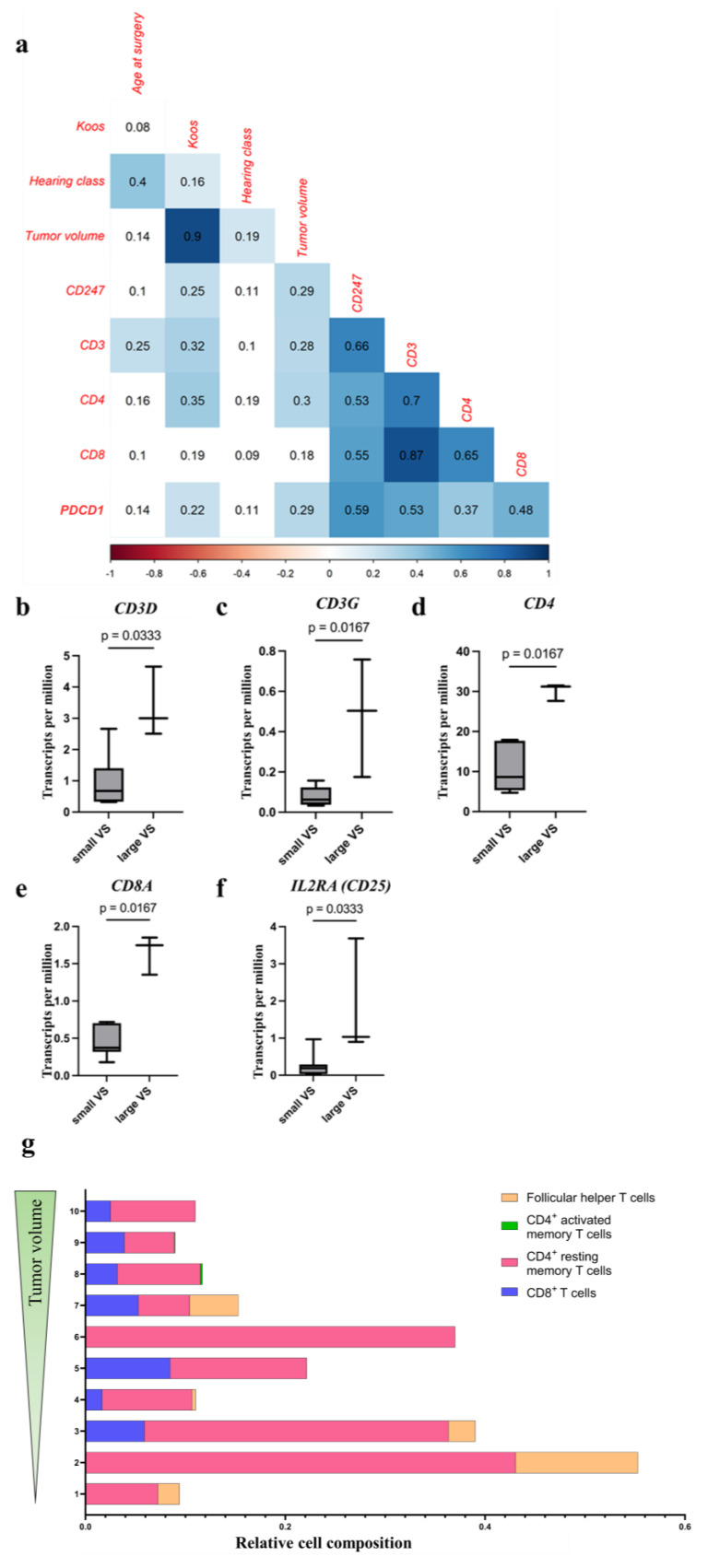
T-cell marker mRNA level and distribution of T-cell subtypes in VS with different tumor volume. Spearman’s rank test correlation of T-cell marker (CD3, CD4, CD8) and inhibitory molecule (CD274, PDCD1) mRNA levels with clinical data (age at surgery, Koos grade, hearing class, and tumor volume) is illustrated. The correlation coefficient r is shown (n = 204). Significant positive correlations (*p* < 0.05) are plotted in blue. Non-significant correlations are plotted uncolored (**a**). The mRNA level of CD3D, CD3G, CD4, CD8A, and IL2RA in VSs of different sizes is illustrated. The differences in the TPM of CD3D (**b**), CD3G (**c**), CD4 (**d**), CD8A (**e**), and IL2RA (**f**) in large and small VSs were calculated using the Mann–Whitney test. Relative cell compositions of CD8^+^ T cells, CD4^+^ resting memory T cells, CD4^+^ activated memory T cells, and follicular helper T cells in VS tumor samples were calculated using deconvolution analysis from bulk RNAseq data. The tumors are sorted by size, with the smallest tumor assigned the number 1 and the largest tumor the number 10 (**g**). Differences in T-cell compositions in small and large VSs are shown in Figure S7.

The gene expression level of *CD3*, *CD4*, *CD247*, and *PDCD1* correlated positively with Koos grade and tumor volume in the analysis of the qPCR data (r = 0.22 to 0.35, *p* < 0.05; Figure 4a; Table S8). In addition, the RNA level of these surface molecules was differentially detected in tumors of different sizes in RNAseq analysis (Figure 4b–f; Tables S27–S31). In large VSs, the RNA level of *CD3D* (small VS, median TPM: 0.68, IQR: 0.95; large VS, median TPM: 3.00, IQR: 1.07, *p* = 0.03; Figure 4b), *CD3G* (small VS, median TPM: 0.06, IQR: 0.08; large VS, median TPM: 0.5, IQR: 0.29, *p* = 0.02; Figure 4c), *CD4* (small VS, median TPM: 8.64, IQR: 8.79; large VS, median TPM: 31.21, IQR: 1.91, *p* = 0.02; Figure 4d), *CD8A* (small VS, median TPM: 0.37, IQR: 0.28; large VS, median TPM: 1.75, IQR: 0.25, *p* = 0.02; Figure 4e), and *IL2RA* (small VS, median TPM: 0.19, IQR: 0.18; large VS, median TPM: 1.03, IQR: 1.39, *p* = 0.03; Figure 4f) were significantly higher than the RNA level in small VSs. The Forkhead-Box-Protein P3 (*FOXP3*) expression level did not differ significantly between large and small VSs (small VS, median TPM: 1.18, IQR: 0.69; large VS, median TPM: 2.51, IQR: 0.26, *p* = 0.18; Figure S7).

The relative cell compositions of various T cells in VSs of different sizes were calculated from the RNAseq data using deconvolution analyses (Figure 4g). The relative cell composition of CD4^+^ resting memory T cells (Figure S8a, Table S32) and follicular helper T cells (Figure S8b, Table S33) tends to be higher in small VSs (CD4^+^ resting memory T cells in small VS, median: 0.14, IQR: 0.26; follicular helper T cells in small VS, median: 0.02, IQR: 0.04) than in large VSs (CD4^+^ resting memory T cells in large VS, median: 0.08, IQR: 0.02; no detected follicular helper T cells in large VS). In contrast, the relative cell composition of CD4^+^ active memory T cells tends to be higher in large VSs (median 0.0009, IQR 0.001) than in small VSs (no detected CD4^+^ active memory T cells) (Figure S8c, Table S34).

#### 3.4.2. Higher Number of T Cells in Larger VSs

**Figure 5:**
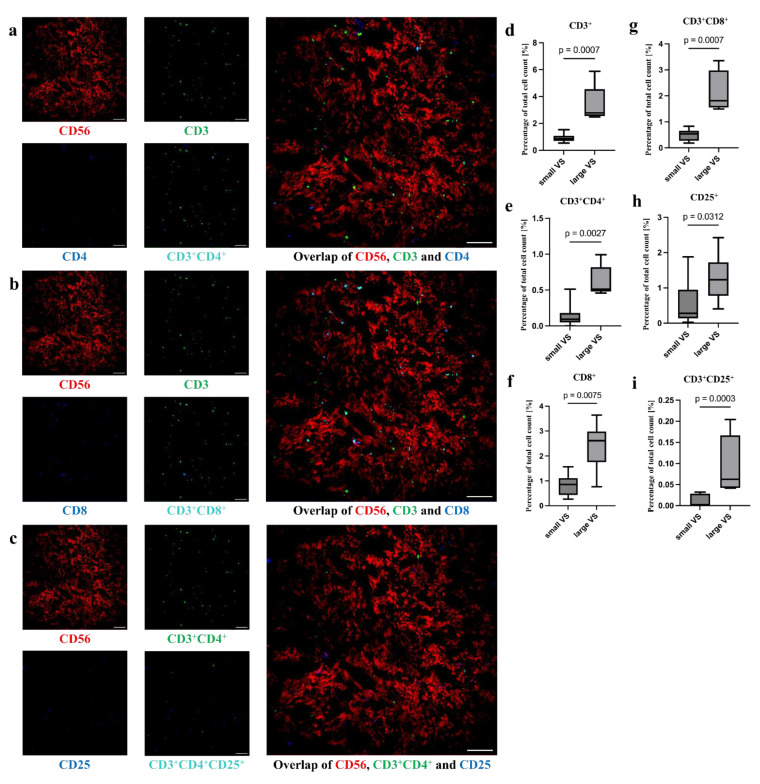
Distribution of different T cells in VSs. CD3^+^, CD4^+^, CD8^+^, and CD25^+^ cells in representative MELC images are shown. The MELC image section has a size of 900 × 900 µm. Fluorescence signals of CD56^+^, CD3^+^, CD4^+^, and CD3^+^CD4^+^ (**a**), CD56^+^, CD3^+^, CD8^+^, and CD3^+^CD8^+^ (**b**) as well as CD56^+^, CD3^+^CD4^+^, CD25^+^, and CD3^+^CD4^+^CD25^+^ cells (**c**) are exemplarily visualized (**a**–**c**). The differences in the percentage of total cell count of CD3^+^ (**d**), CD3^+^CD4^+^ (**e**), CD8^+^ (**f**), CD3^+^CD8^+^ (**g**), CD25^+^ (**h**), and CD3^+^CD25^+^ cells (**i**) between small and large VSs were calculated using the Mann–Whitney test (**d**–**i**). Scale bar = 100 µm (**a**–**c**).

In the VS tumor tissue, a low number CD3^+^ cells were visible (Figure 5). These cells were also positive for CD4, CD8, or CD25 (Figure 5a,b). The CD3^+^CD8^+^ cells were usually distributed over the entire image section, although there was an accumulation around the CD31^+^ blood vessels. CD3^+^CD4^+^ cells were found almost continuously in the vicinity of CD31^+^ blood vessels. The distribution of CD3^+^CD4^+^CD25^+^ cells was difficult to evaluate as the number of cells present was very low (Figure 5c). In large tumors, the percentage of CD3^+^ cells (median small tumors 0.86%, IQR 0.26; median large tumors 2.78%, IQR 0.44, *p* < 0.001; Figure 5d, Table S35), CD3^+^CD4^+^ cells (small tumors, median: 0.09%, IQR: 0.09; large tumors, median: 0.51%, IQR: 0.11, *p* = 0.002; Figure 5e, Table S36), CD8^+^ cells (small tumors: 0.86%, IQR: 0.52; large tumors, median: 2.61, IQR: 0.37, *p* = 0.01; Figure 5f, Table S37), CD3^+^CD8^+^ cells (small tumors, median: 0.53%, IQR: 0.35; large tumors, median: 1.81%, IQR: 0.75, *p* < 0.001; Figure 5g, Table S38), CD25^+^ cells (small tumors, median: 0.28%, IQR: 0.73; large tumors, median: 1.23%, IQR: 0.37, *p* = 0.03; Figure 5h, Table S39), and CD3^+^CD25^+^ cells (small tumors, median: 0.00%, IQR: 0.03; large tumors, median: 0.06%, IQR: 0.06, *p* < 0.001; Figure 5i, Table S40) was higher compared to small VSs.

#### 3.4.3. Higher Amount of CD279 on T_c_ Cells, and CTLA4 Level in Large Tumors

**Figure 6:**
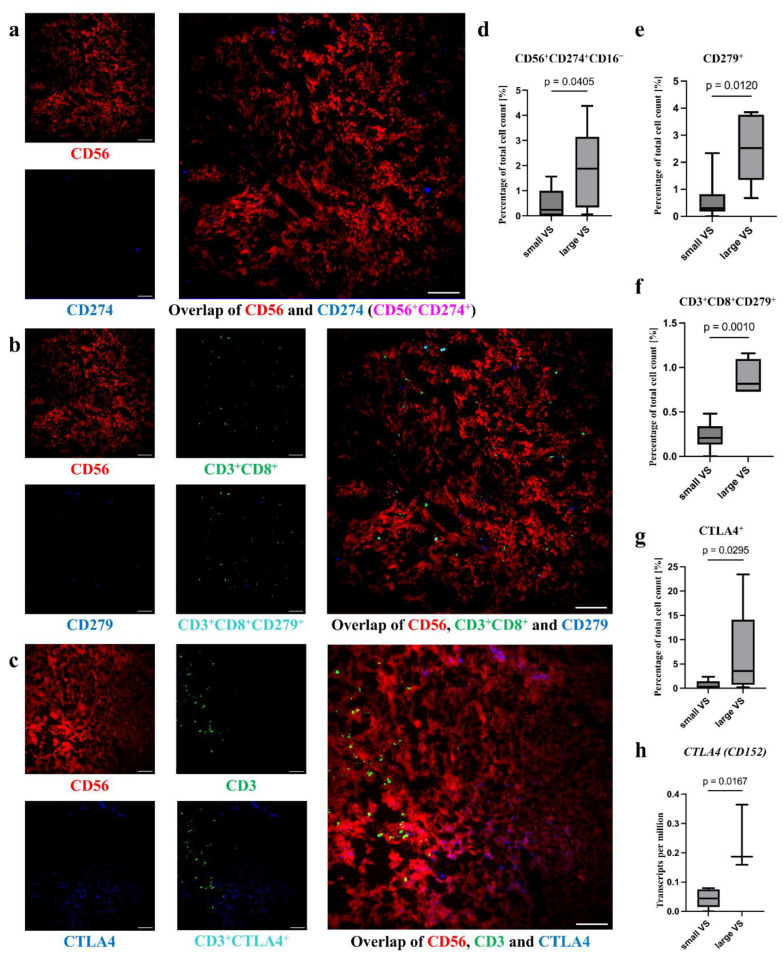
Distribution of T cells with inhibitory receptors (CD274, CD279, CTLA4). CD274^+^, CD279^+^, and CTLA4^+^ cells in MELC images of different sized VSs are visualized. The MELC image section has a size of 900 × 900 µm. CD274^+^ and CD56^+^CD274^+^ cells (**a**) and CD279^+^ and CD3^+^CD8^+^CD279^+^ (**b**), as well as CTLA4^+^ and CD3^+^ CTLA4^+^ cells (**c**) are illustrated in the MELC images. The differences between small and large VSs of CD16^−^CD56^+^CD274^+^ (**d**), CD279^+^ (**e**), CD3^+^CD8^+^CD279^+^ (**f**), and CTLA4^+^ (**g**) cells were calculated using Mann–Whitney test. In addition, RNA levels of *CTLA4* in 10 VSs of different sizes are analyzed (**h**). Differences between large and small tumors in mRNA expression were determined by Mann–Whitney test. Scale bar = 100 µm (**a**–**c**).

The surface molecules CD274, CD279, and CTLA4 were detected in VS tumor tissue (Figure 6a–c). The signal for CD274^+^ and CTLA4^+^ cells was very low and showed low association with other analyzed surface molecules. The amount of CD3^+^CD8^+^CD279^+^ cells in the total T_c_ cell count showed no difference between large and small VSs (small tumors, median: 39.13%, IQR: 25.69; large tumors, median: 48.57%, IQR: 16.23, *p* > 0.999; Figure S9, Table S41). However, more CD56^+^CD274^+^CD16^-^ tumor cells (small tumors, median: 0.24%, IQR: 0.87; large tumors, median: 1.88%, IQR: 1.92, *p* = 0.041) were detected in large tumors than in small tumors (Table S42, Figure 6d).

It should be noted that most of the CD279 signals originate from cells that were also CD3^+^ cells. An association of CD279 with the CD3 signal was observed. The percentage of CD279^+^ cells (small tumors, median: 0.30%, IQR: 0.51; large tumors, median: 2.53%, IQR: 1.37, *p* = 0.01; Figure 6e, Table S43) and CD3^+^CD8^+^CD279^+^ cells (small tumors, median: 0.21%, IQR: 0.05; large tumors, median: 0.82%, IQR: 0.23, *p* = 0.001; Figure 6f, Table S44) was higher in large VSs than in small. In addition to the proportion of CD279^+^ T_c_ cells to total cell count, the proportion of CD279^+^ T_c_ cells to T_c_ cell count was calculated in small and large VSs.

CTLA4^+^ cells (small tumors, median: 0.19%, IQR: 0.59; large tumors, median: 3.57%, IQR: 2.66, *p* = 0.03; Figure 6f, Table S45) were more frequently detected in large tumors than in small tumors. Additionally, in RNAseq analysis, a differential RNA level of *CTLA4* for large and small tumors was detected (Figure 6g, Table S46). The RNA level was higher for large tumors (median TPM: 0.19, IQR: 0.10) than for small tumors (median TPM: 0.04, IQR: 0.04, *p* = 0.02).

## 4. Discussion

The interaction with immune cells plays an important role in tumor progression and cancer-cell proliferation [5]. Both macrophages and T cells influence the progression of numerous tumors [6]. Different studies have been conducted to investigate the relationship between clinical symptoms and tumor-tissue inflammation triggered by CD45, as well as the correlation between CD45 expression, tumor size, and tumor growth index in VS [31,32]. Gregory et al. described a tumor milieu with more immune cells in growing- than in static VS [33]. In our study, the expression of CD45 in histological sections and mRNA levels in both transcriptome analyses and qPCR data were positively associated with tumor volume. These findings were consistent with other studies, as macrophages and lymphocytes are also associated with a higher tumor volume [3,4,19,34].

Numerous studies investigated macrophages in VSs: According to Gregory et al., macrophages were the most abundant immune-cell population in VSs, accounting for approximately one-third of the cell mass [35]. Macrophage infiltration of up to 50% of tumor-infiltrating cells has been described for malignant tumors [36]. Similarly, in single-cell analyses conducted by Barrett et al., the infiltration of myeloid cells was higher in large VSs than in small VSs [37]. In our study, macrophage infiltration in large VSs amounted to a median of 20.51% compared to a median of 7.42% of the cell count in small VSs, with a maximum infiltration of 38.00% of the total cell count. Additionally, relative cell composition of macrophages in the deconvolution analysis was higher in large VSs than in small VSs, and relative cell composition of monocytes and inflammatory and anti-inflammatory macrophages tended to be higher in large VSs compared to small VSs. In many studies, the macrophage markers CD68 and CD163 correlated positively with the tumor volume and growth rate of VSs [3,19,32,34,38,39]. TAMs expressing those markers were associated with a shorter overall survival of the patients in some malignant tumors and have tumor progression-promoting properties. Gregory et al. showed that growing VSs had a higher number of alternatively activated macrophages than static VSs [33]. In the study by Baruah et al., a positive correlation between CD14 expression and tumor volume was demonstrated [40]. To date, there have been no studies on the expression of CD16 and CD40 in VSs. In our study, the expression of CD68 in histological sections and the mRNA level in transcriptome analysis, the expression of CD163 in histological sections, and the number of CD68^+^CD163^+^ cells were positively associated with tumor size. Although the percentage of CD14^+^, CD68^+^CD14^+^, CD16^+^, and CD68^+^CD16^+^ cells in the histological sections showed no association with the tumor volume, there was a positive association of the mRNA levels in the transcriptome analysis and qPCR with the tumor volume. Monocytes can produce tumoricidal substances, on the one hand, and differentiate into TAMs, on the other. In contrast, CD40^+^ and CD68^+^CD40^+^ cells showed a positive association with tumor volume in the quantification of histological sections, but no significant results in mRNA expression. However, the percentage of CD40^+^ cells in the total cell count was very low. These M1 macrophages promote anti-tumor response. To date, only one study has investigated not only the presence of macrophages and their correlation with tumor volume and growth rate, but also the spatial distribution within the tumor. Nickl et al. described the distribution of CD68^+^ cells throughout the tumor area, which is also demonstrated by our data [41]. Furthermore, the authors noted that CD14^+^ and CD163^+^ cells are mainly found in regions with a low cell density. Macrophages, however, were mostly distributed throughout the tumor area. This distribution throughout the tumor could be due to the presence of tissue-resident macrophages, which are found in every tissue in a healthy organism [42]. For malignant tumors, the possibility of TAMs arising from both tissue-resident macrophages and circulating monocytes has been described [43]. The tumor macrophages could develop from these macrophages and attract further macrophages, e.g., by cytokine secretion from the blood or the environment. Individual cell populations (in some tissue sections CD68^+^ and CD14^+^ cells) showed a clustering around blood vessels. This may suggest that the CD14^+^ cells were monocytes that have migrated from the blood. These could differentiate further into CD68^+^ macrophages. In addition, a co-occurrence of CD16^+^ and CD163^+^ cells were observed in our study. The literature described CD163 expression in CD14^+^CD16^+^ monocytes [44]. As soon as the expression of CD163 on monocytes increases, they differentiate into M2 macrophages [45]. It could, therefore, be possible that the migrated CD16^+^ monocytes differentiate into M2 macrophages and, thus, a co-occurrence of CD16 and CD163 could occur.

The literature described significantly fewer T cells than macrophages in VSs. However, both CD4^+^ and CD8^+^ T cells could be detected in the tumors. In the study by Huo et al., single-cell analysis revealed a higher number of lymphocytes in VSs compared to the tissue of non-tumorous auditory nerves [46]. Bi et al. described an association between tumor volume in the VS and the presence of CD4, but not with the presence of CD8 [34]. In other studies, a significant enrichment of CD4^+^ and CD8^+^ T cells was found especially in large and fast-growing tumors [34,38,39]. In our study, T_c_ cells, T_H_ cells, and T_reg_ cells were detected in histological sections of VSs. Similarly, to the established literature, the number of T cells was very low. The percentage of CD3^+^ cells in the total number of cells in histological sections and the mRNA expression in a transcriptome analysis and in qPCR were higher in large tumors than in small tumors. Additionally, the percentages of CD3^+^CD4^+^, CD3^+^CD8^+^, and CD3^+^CD25^+^ T cells were higher in large tumors compared to small tumors. Furthermore, the mRNA expression of *CD4*, *CD8A*, and *CD25* was higher in larger tumors than in small VSs. In the deconvolution analysis, the relative cell composition of CD4^+^ active memory T cells tends to be higher in large VSs than in small VSs. In contrast, the relative cell composition of CD4^+^ resting memory T cells and follicular helper T cells tended to be higher in small VSs than in large VSs. In malignant tumors, T_c_ cells detect tumor cells via surface antigens and, thus, ensure an anti-tumor immune response via destruction of tumor cells. T_H_ cells can exhibit anti-tumoral effects (T_H1_ cells) as well as pro-tumoral functions (T_H2_ cells) in malignant tumors [6]. However, T_reg_ cells inhibit the anti-tumoral immune response in cancer. An accumulation of T_c_ cells in cell-poor regions was described in the study by Nickl et al. [41]. In our study, T_c_ cells were distributed throughout the tumor and a regional association between T_H_ cells and blood vessels was observed. Recent studies have investigated the presence of tissue-resident CD8^+^ memory T cells [47]. These were distributed in the tumor and were responsible for anti-tumor defense. The T_c_ cells detected could be tissue-resident CD8^+^ memory T cells distributed in the tumor. The minority of T cells in the VSs expressed CD25, a surface protein that is highly expressed on activated T cells [48]. It could, therefore, mean that the T cells found were in the tumor but were inactive. The T_H_ cells, on the one hand, could be associated with the blood vessels, as these potentially migrate into the tumor. On the other hand, the low number of T_H_ cells could also lead to a falsely assumed association.

An increasing number of studies are focusing on possible immunomodulation in VSs, with CD274 in particular being the focus of recent research. Some studies described the presence of CD279 and CD274 in sporadic VS and VS in patients with NF2-associated VS [33,39,49,50]. The expression of CD274 was detected in both static and growing VS, whereby Bi et al. detected an association between the growth rate and the presence of CD274 [34]. In a study by Lee et al., 57% of tumors were CD274^+^ [7]. An increased expression of the CD279 and CTLA4 signaling pathways was detected in VSs with an increased number of immune cells [51]. In one case report, a patient benefited from the postoperative administration of pembrolizumab, a CD279 inhibitor. Under the medication, the postoperative growth of the VS stagnated [52]. Our results showed a weak expression of CD274, CD279, and CTLA4 in VS. However, the histological sections revealed an association of CD274^+^ tumor cells with tumor volume. Furthermore, there was a positive association between the tumor volume of VS and the expression of CD279, CD279^+^ T_c_ cells, and CTLA4 in histological sections. The transcriptome analysis also showed a positive association between *CTLA4* and the tumor volume of VS and the *PDCD1* level correlated positively with the tumor volume in qPCR analysis.

In our cohort of VSs, as in established studies, an increased number of different immune cells was found in larger VSs [3,4,19,34]. In addition to the frequently investigated CD68^+^ and CD163^+^ macrophages, other macrophage populations were detected in our study, which may influence VS progression. It is conceivable that the detected monocytes develop into the existing M1 and M2 macrophages or that the monocytes also influence the progression of VS. However, these circumstances have not yet been investigated and should be the subject of further research. Single-cell analyses of VS revealed that immune cells represent the proliferative cell population within the tumor [37,46]. The proliferation of immune cells could promote greater tumor volume via the mass effect. The data also showed an association between T cells and VS tumor volume and growth rate [4,38,39]. However, little is currently known about the mechanisms by which these immune-cell populations influence VS and its progression. Our data may suggest that T_c_ cells attempt to influence VS progression via immunomodulation through the CD274-CD279 axis, as many of the T_c_ cells express CD274 (39.13% in small VS and 48.57% in large VS). The results of other studies also support this hypothesis [51,52,53]. Some of the tumor cells inhibit apoptosis induction by T cells via expression of CD279, which interacts with CD274 [7,33,39]. In addition, the expression of CTLA4 was increased in large tumors. Through the inhibition of T-cell proliferation, this could also contribute to immune escape, particularly in large VSs [15]. However, the exact mechanisms behind this influence are unknown and must be further investigated. Furthermore, the strong immune-cell infiltration, especially in large tumors, offers possible starting points for the investigation and establishment of drug treatment options. The effect of already established drugs on the progression of VSs could be investigated. The CD274-CD279 axis is particularly promising, as the efficacy of the CD279 inhibitor pembrolizumab was confirmed in one patient in a case report [52].

The data suggested a higher number of macrophages, which are cells of the innate immune defense, compared with T cells, which are components of the adaptive immune system, in VSs. This is consistent with a recent single-cell analysis of VS. In the study of Dong et al., a greater number of macrophages compared to T cells was also detected in the tumor [54]. In malignant tumors, a higher number of T cells was identified in the stroma around the tumors whereby the number of T cells inside the tumor was positively associated with the number of T cells outside the tumor [55]. For the VSs, it is difficult to examine the stroma, as it is the vestibulocochlear nerve, which should be protected as much as possible during tumor removal. This may be due to the function of the cells, as macrophages do not have a specific target but instead recognize patterns on the surface of cells [56], whereas T cells are directed against specific surface structures on the target cells [57]. Therefore, the desired effect might require fewer T cells than macrophages to achieve a similar result. However, M2 macrophages also have increased tumor growth-promoting properties and promote tumor progression [58,59]. It would, therefore, promote VS progression to have a particularly large number of M2 macrophages, which have a positive influence on progression, rather than other immune cells that could potentially prevent and contain growth and progression. The properties of other immune cells, such as T cells, have not yet been investigated in detail in VSs. In addition to macrophages, T cells showed a positive association with tumor volume. The influence of T cells on tumor progression or the inhibition of VS tumor progression has not yet been clarified. Naturally, T_c_ cells tend to contribute to tumor defense. T_reg_ cells, on the other hand, tend to inhibit the effector response of other T cells and would, thus, promote tumor progression. Additionally, in malignant tumors, a reduction in the anti-tumor activity of T cells induced by TAMs has been described. The phenotype of T_c_ cells in malignant tumors is described as dysfunctional [6].

The number of samples used in the MELC analyses is very small. The methods for the spatial analysis of several different antigens and, thus, different cell populations in a single tissue section are still largely unknown and not widely used. Besides MELC, there are many different ways to determine the spatial distribution of different cell populations [17]. In order to achieve a higher level of significance, a larger number of samples is required.

## 5. Conclusions

In summary, our data suggested the presence of macrophages and T cells in the VSs, in line with the literature. In our study, many M2 macrophages were detected, particularly in large VSs. Additionally, monocytes and a few M1 macrophages were found in the VSs. Compared to macrophages, T cells were present in significantly lower numbers in the VSs. The T cells can be subdivided into T_c_ cells, T_H_, and T_reg_ cells, all of which are particularly abundant in large VSs. Our data also suggest a possible immunomodulatory component in the VS, as CD279^+^ (PD-1^+^) T_c_ cells, as well as CTLA4 and CD274 (PD-L1) expression were detected in the VSs.

## Data Availability

The raw data and meta data of RNAseq analysis can be accessed in the Sequence Read Archive (SRA) of the National Center for Biotechnology (BioProject ID: PRJNA1150102). Further original contributions presented in the study are included in the article and Supplementary Materials, further inquiries can be directed to the corresponding author.

## Supplementary Materials

The following supporting information can be downloaded at https://www.mdpi.com/article/10.3390/cancers18030355/s1, Figure S1: Flowchart of the study; Figure S2: Output images CellProfiler of the *IdentifyPrimaryObjects* Tool; Figure S3: Image of representative CD56 and S100 staining of VS cryosection tissue; Figure S4: Exemplary representation of CD31^+^ and CD56^+^ cells in a MELC image; Figure S5: mRNA expression of *CD14* and *CD163* is illustrated in 10 VSs of different sizes; Figure S6: Analysis of CD14^+^, CD16^+^, CD68^+^, and CD68^+^ cells in MELC images of 5 VSs; Figure S7: mRNA level of *FOXP3* in VS. The differences of mRNA level were calculated by Mann–Whitney test; Figure S8: Deconvolution analysis of bulk RNAseq data; Figure S9: Quantification of CD274^+^ Tc cells from MELC images of different sized VS; Table S1: Baseline data of 204 patients with VS used for qPCR analysis.; Table S2: Homo sapiens specific primers used for quantitative Real-Time PCR; Table S3: Baseline data of 10 patients with VS used for RNAseq analysis; Table S4: Baseline data of 5 patients with VS used for MELC analysis; Supplemental method S1: Preparation of cryosections; Supplemental method S2: Immunohistochemical staining; Table S5: Antibody used for immunohistochemistry; Table S6: Antibodies used for MELC; Table S7: Classification of VS developed by Koos; Table S8: Mean expression of CD56 in VS samples used for MELC; Table S9. Spearman’s r and *p* values for correlation analysis of investigated surface markers and clinical parameters in 204 patients with sporadic VS; Table S10: Mann–Whitney test of *PTPRC* RNA level in VS tumor samples; Table S11: Mann–Whitney test of CD45 expression detected in MELC images in large and small VS; Table S12: Mann–Whitney test of *CD14* RNA level in VS tumor samples; Table S13: Mann–Whitney test of *FCGR3A* (CD16) RNA level in VS tumor samples; Table S14: Mann–Whitney test of *CD68* RNA level in VS tumor samples; Table S15: Mann–Whitney test of *CD163* RNA level in VS tumor samples; Table S16: Mann–Whitney test of relative total macrophage composition in VS tumor samples; Table S17: Mann–Whitney test of relative monocyte composition in VS tumor samples; Table S18: Mann–Whitney test of relative M1 macrophage composition in VS tumor samples; Table S19: Mann–Whitney test of relative M2 macrophage composition in VS tumor samples; Table S20: Mann–Whitney test of CD68 expression detected in MELC images in large and small VSs; Table S21: Mann–Whitney test of CD40 expression detected in MELC images in large and small VSs; Table S22: Mann–Whitney test of CD163 expression detected in MELC images in large and small VSs; Table S23: Mann–Whitney test of CD40^+^CD68^+^ expression detected in MELC images in large and small VSs; Table S24: Mann–Whitney test of CD163^+^CD68^+^ expression detected in MELC images in large and small VSs; Table S25: Mann–Whitney test of CD14^+^CD68^+^ expression detected in MELC images in large and small VSs; Table S26: Mann–Whitney test of CD16^+^CD68^+^ expression detected in MELC images in large and small VSs; Table S27: Mann–Whitney test of *CD3D* RNA level in VS tumor samples; Table S28: Mann–Whitney test of *CD3G* RNA level in VS tumor samples; Table S29: Mann–Whitney test of *CD4* RNA level in VS tumor samples; Table S30: Mann–Whitney test of *CD8A* RNA level in VS tumor samples; Table S31: Mann–Whitney test of *IL2RA* (CD25) RNA level in VS tumor samples; Table S32: Mann–Whitney test of relative CD4^+^ resting memory T-cell composition in VS tumor samples; Table S33: Mann–Whitney test of relative follicular helper T-cell composition in VS tumor samples; Table S34: Mann–Whitney test of relative CD4^+^ active memory T-cell composition in VS tumor samples; Table S35: Mann–Whitney test of CD3 expression detected in MELC images in large and small VS; Table S36: Mann–Whitney test of CD3^+^CD4^+^ expression detected in MELC images in large and small VS; Table S37: Mann–Whitney test of CD8 expression detected in MELC images in large and small VS; Table S38: Mann–Whitney test of CD3^+^CD8^+^ expression detected in MELC images in large and small VS; Table S39: Mann–Whitney test of CD25 expression detected in MELC images in large and small VS; Table S40: Mann–Whitney test of CD3^+^CD25^+^ expression detected in MELC images in large and small VS; Table S41: Mann–Whitney test of the proportion of CD274^+^ T_c_ cells in the total T_c_ cell count detected in MELC images in large and small VS; Table S42: Mann–Whitney test of CD56^+^CD274^+^CD16^-^ expression detected in MELC images in large and small VS; Table S43: Mann–Whitney test of CD279 expression detected in MELC images in large and small VS; Table S44: Mann–Whitney test of CD3^+^CD8^+^CD279^+^ expression detected in MELC images in large and small VS; Table S45: Mann–Whitney test of CTLA4^+^ cells detected in MELC images in large and small VS; Table S46: Mann–Whitney test of *CTLA4* (CD152) RNA level in VS tumor samples.

## Abbreviations

The following abbreviations are used in this manuscript:

AAO-HNS	American Academy of Otolaryngology- Head and Neck Surgery
CTLA-4	Cytotoxic T-lymphocyte-associated protein 4
FCGR3A	F_c_ gamma receptor III
IHC	immunohistochemical staining
IL2RA	Interleukin-2 receptor alpha chain
LCA	Leukocyte Common Antigen
MELC	Multi-epitope-ligand cartography
MRI	magnetic resonance imaging
PDCD1/PD-1	Programmed Cell Death Protein 1
PD-L1	Programmed Death-Ligand 1
PTPRC	protein tyrosine phosphatase receptor type C
qPCR	quantitative real-time polymerase chain reaction
SD	Surditas for deafness
TAMs	tumor-associated macrophages
T_c_ cells	Cytotoxic T cells
T_H_ cells	T-helper cells
T_reg_ cells	Regulatory T cells
VS	Vestibular schwannoma
