# Supplementary material for: Composition of Immune Cells in Sporadic Vestibular Schwannomas with Different Tumor Volumes
Source: Cancers (Basel). 2026 Jan 23;18(3):355. doi: 10.3390/cancers18030355 (PMC12897149; doi:10.3390/cancers18030355)
Supplement: Supplementary file 1 [file cancers-18-00355-s001.zip › cancers-4063715-Supplementary.pdf]

cancers

Composition of Immune Cells in Sporadic Vestibular Schwannomas with Different Tumor Volumes

Anna-Louisa Becker [1], Clara Helene Klause [1], Martin Sebastian Staege [2], Edith Willscher [3], Jonas Scheffler [4], Paola Schildhauer [1], Christian Ostalecki [5], Christian Strauss [1], Julian Prell [1], Christian Scheller [1], Stefan Rampp [1,6,7] and Sandra Leisz [1,*]

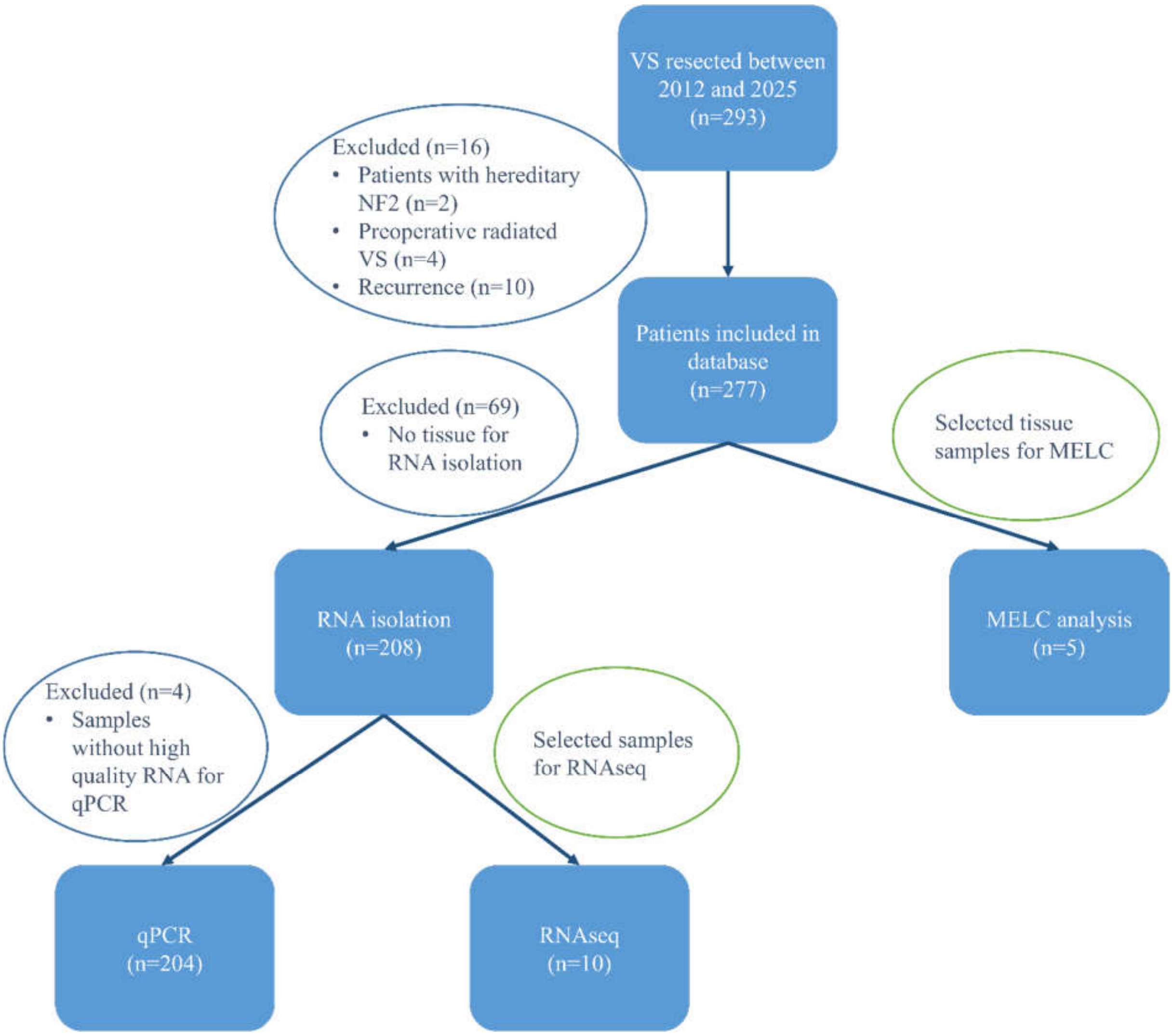

Figure S1. Flowchart of study.

 https://doi.org/10.3390/cancers18030355

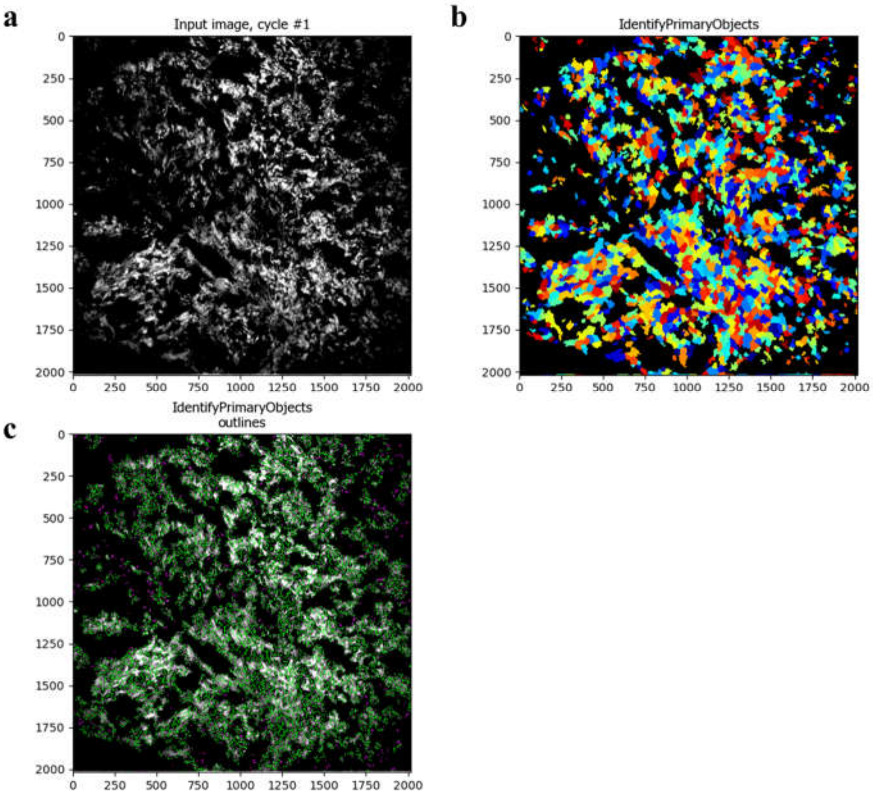

Figure S2. Output images CellProfiler of the *IdentifyPrimaryObjects* Tool. The image shows both the original image in which the objects are to be identified (a) and the images after the objects have been calculated (b and c). Firstly, the objects are displayed in different colors (b). Secondly, the boundaries of the identified objects are shown (c). The counted objects are shown in green and the objects that were not counted due to their incorrect size are shown in red.

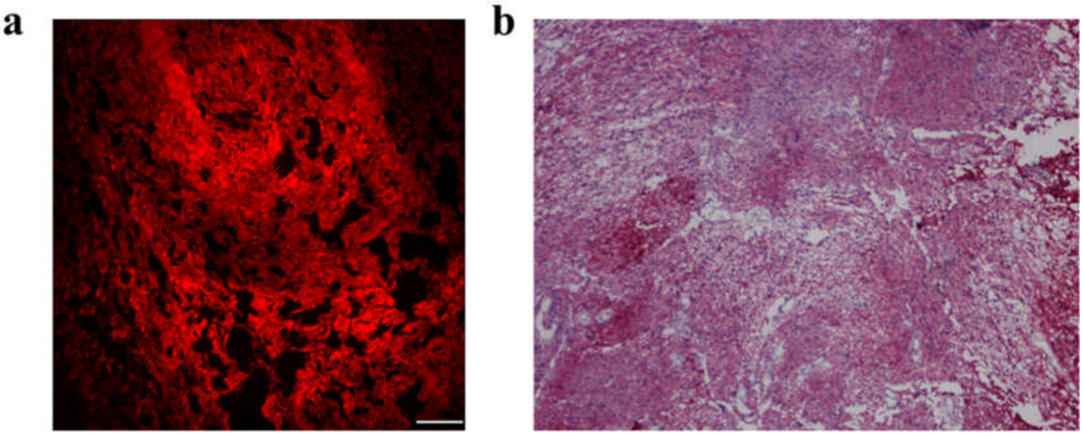

Figure S3. Image of representative CD56 and S100 staining of VS cryosection tissue. Fluorescence image of a VS cryosection stained with CD56 for MELC. Scale bar represent 100 µm (a). IHC image of a VS cryosection at 4x magnification, stained with a red chromogen (b).

https://doi.org/10.3390/cancers18030355

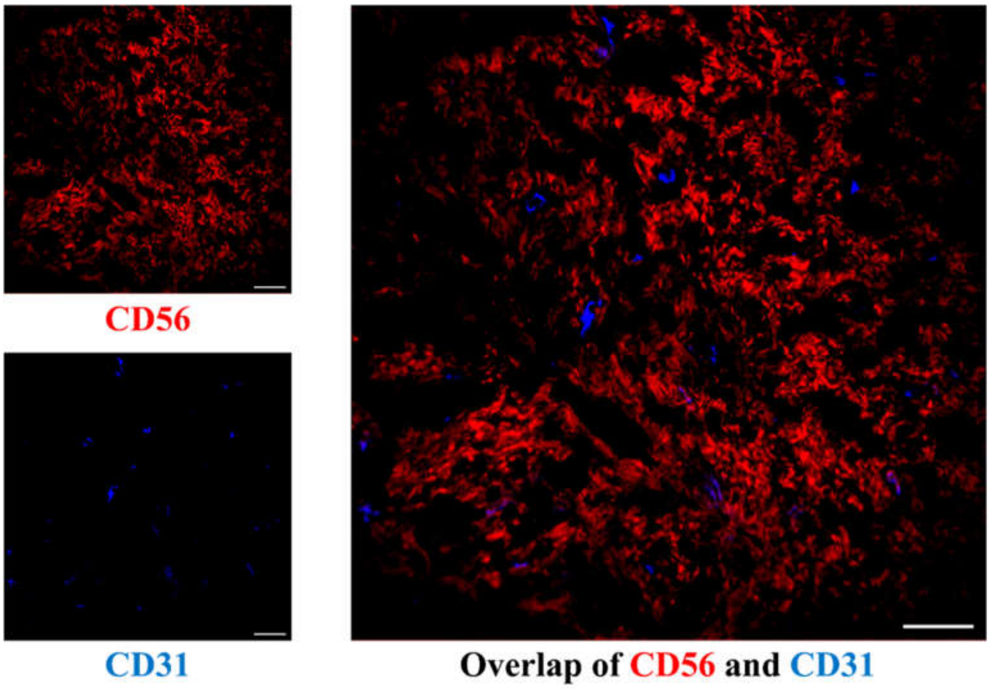

Figure S4. Exemplary representation of CD31⁺ and CD56⁺ cells in a MELC image. The scale bar represents 100 μm.

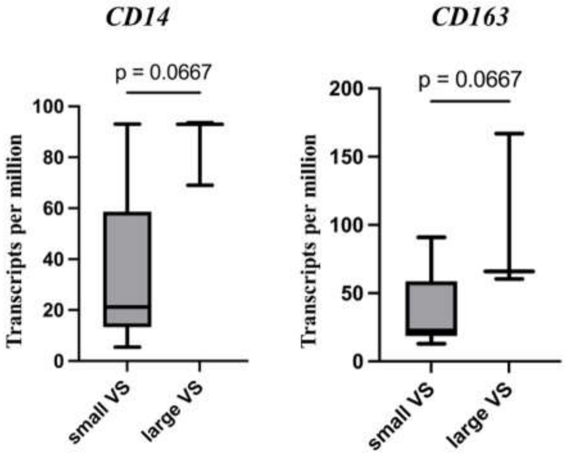

Figure S5. mRNA expression of *CD14* and *CD163* is illustrated in 10 VS of different sizes. The differences of mRNA level were calculated by Mann Whitney test.

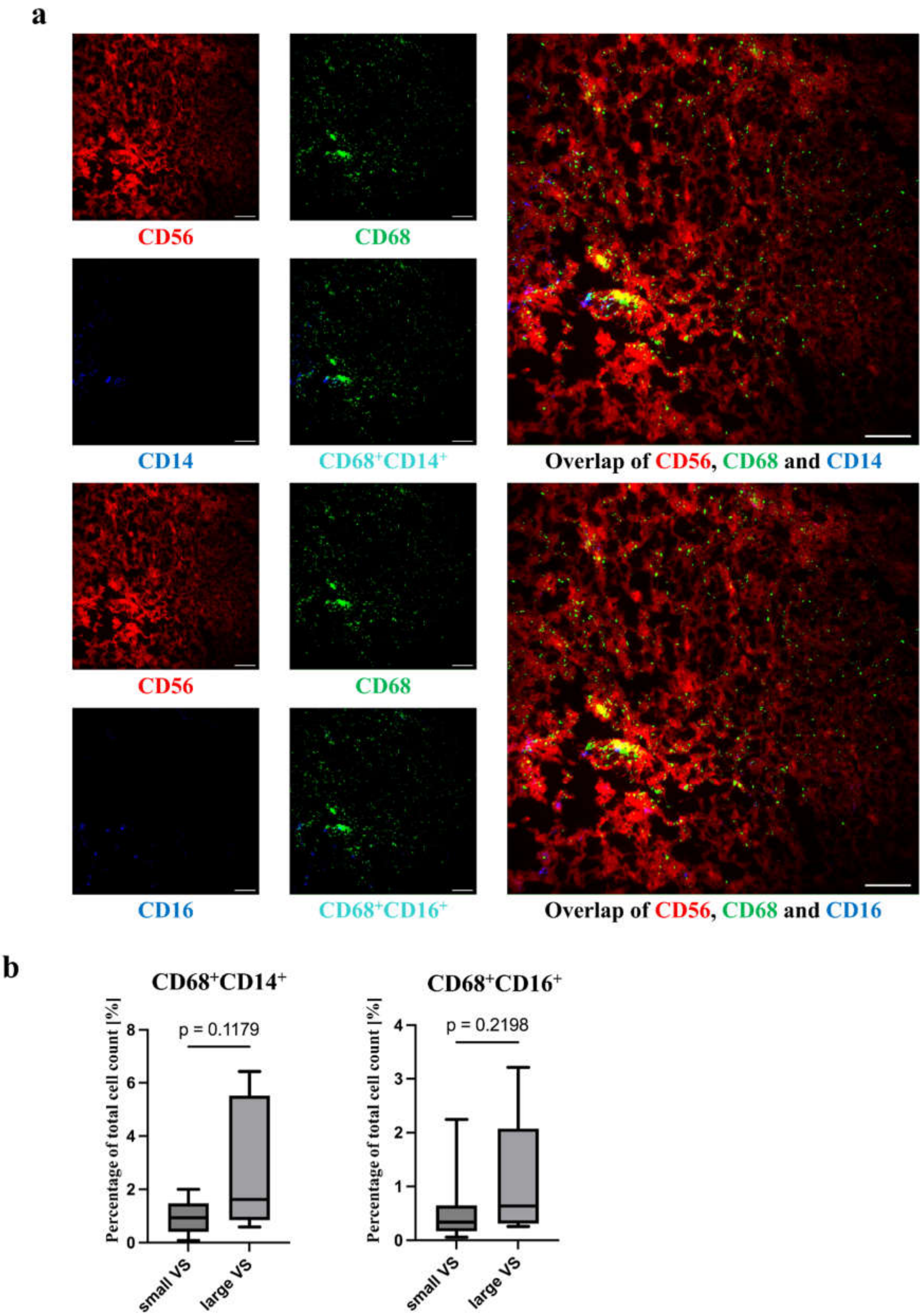

Figure S6. Analysis of $CD14^+$, $CD16^+$, $CD68^+$and $CD68^+$ cells in MELC images of 5 VS. The MELC image section has a size of 900x900µm. Fluorescence signals of CD14, CD16, CD56 and CD68, as well as $CD68^+CD14^+$ cells and $CD68^+CD16^+$ cells are visualized (a). In addition, the difference in the percentage of $CD68^+CD14^+$ and $CD68^+CD16^+$ cells to the total cell count in small and large VS was calculated using the Mann Whitney test (b).

https://doi.org/10.3390/cancers18030355

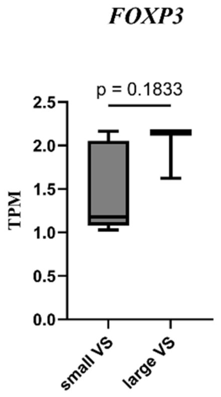

Figure S7. mRNA level of *FOXP3* in VS. The differences of mRNA level were calculated by Mann Whitney test.

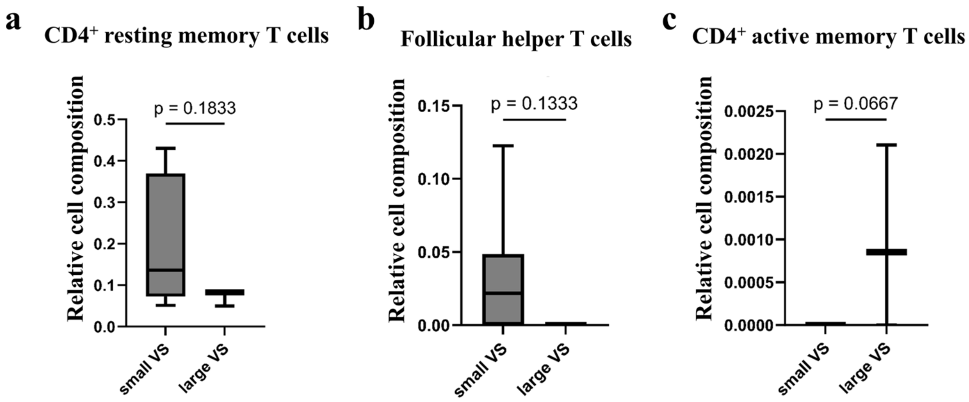

Figure S8. Deconvolution analysis of bulk RNAseq data. Differences of relative cell composition in small and large VS of CD4+ resting memory T cells (a) follicular helper T cells (b) and CD4+ active memory T cells (c).

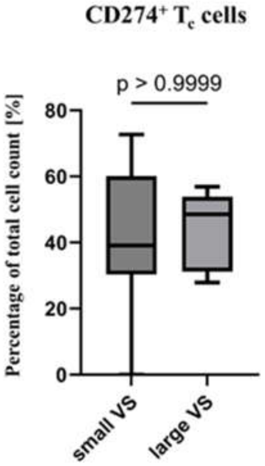

Figure S9. Quantification of CD274+ Tc cells from MELC images of different sized VS. Statistical difference was calculated using Mann Whitney test.

Table S1. Baseline data of 204 patients with VS used for qPCR analysis.

Variable	Total (n=204)
Male	88
Female	116

Mean age (years) and standard deviation (SD)	51 ± 12.52 (range:18 - 77)
Median age (years) and interquartile range (IQR)	52 (16.25)
Koos grade	
1	4
2	67
3	68
4	63
Without Koos grade	2
AAO-HNS[1] (hearing class)	
A	59
B	53
C	34
D	26
DS[2]	17
Without preoperative hearing class	15
Mean tumor volume (cm^3) and SD	3.98 ± 5.23 (range: 0.10 – 37.03)
Median tumor volume (cm^3) and IQR	2.09 (4.47)

[1] AAO-HNS: American Academy of Otolaryngology-Head and Neck Surgery.

Table S2. Homo sapiens specific primers used for quantitative Real-Time PCR.

Gene symbol (protein name)	Primer sequence 5′ to 3′ (forward, reverse)	Annealing temperature (°C)	Product length	Reference sequence
CD68	CGCAGCACAGTGGACATTCT, GGATCAGGCCGATGATGAGAG	60<br>60	236	NM_001251.3
CD163	AAAAAGCCACAACAGGTCGC, ATGGCCTCCTTTTCCATTCCA	60<br>60	322	NM_004244.5
CD4	CAGGAAGTGAACCTGGTGGT, CTCAGCAGA-CACTGCCACAT	60<br>60	191	NM_000616.5
CD8	CCTGAGCAACTCCATCATGT, AGGA-GAAGGACCCCACAAGT	58<br>60	243	NM_001768.7
CD3	TGGTACTGGCTACCCTTCTCTC, CCCAGGTCCAG-TCTTGTAATGT	60<br>60	154	NM_000732.6
CD40	GGTGAGTGACTGCACAGAGTTC, GCAGATGGTGTCTGTTTCTGAG	61<br>59	169	NM_001250.6
PTPRC (CD45)	CTCTACGCAAAGCTAGGCCA, TGACAGAATGTTCTGGCCCC	60<br>60	276	NM_002838.5
CD14	CCTAAAGGACTGCCAGCCAA GGAGTTCATTGAGCCCTCGT	60<br>60	165	NM_000591.4
FCGR3A (CD16)	ACACTCAAAGACAGCGGCTC AGTCCTGTGTCCACTGCAAA	60<br>60	188	NM_000569.8
CD247	CGCAGAGAAGGAAGAACCCTC, CATGTGAAGGGCGTCGTAGG	60<br>61	176	NM_198053.3
PDCD1 (CD279)	TGACTTCCACATGAGCGTGG, TGGCTCCTATTGTCCCTCGT	60<br>60	296	NM_005018.3
GAPDH	TCGTGGAAGGACTCATGACC, TTCCCGTTCAGCTCAGGGAT	60<br>60	172	NM_002046.7

Table S3. Baseline data of 10 patients with VS used for RNAseq analysis.

Variable	Small VS (n=7)	Large VS (n=3)
Male	4	1
Female	3	2
Mean age (years) and standard deviation (SD)	46 ± 6.72 (range: 33-57)	57.67 ± 10.78 (range: 46-72)

https://doi.org/10.3390/cancers18030355

Median age (years) and interquartile range (IQR)	46 (5)	55 (13)
Koos grade		
1	1	0
2	1	0
3	5	1
4	0	2
AAO-HNS[1] (hearing class)		
A	2	1
B	3	0
C	2	2
D	0	0
DS[2]	0	1
Mean tumor volume (cm^3) and SD	1.23 ± 0.98 (0.18 – 2.86)	5.7 ± 10.78 (5.13 - 6.83)
Median tumor volume (cm^3) and IQR	1.00 (1.42)	5.1 (0.85)

[1] AAO-HNS: American Academy of Otolaryngology-Head and Neck Surgery.

Table S4. Baseline data of 5 patients with VS used for MELC analysis.

Variable	Total (n=5)
Male	3
Female	2
Mean age (years) and standard deviation (SD)	50 ± (43-62)
Median age (years) and interquartile range (IQR)	48 (6)
Koos grade	
1	0
2	2
3	1
4	2
AAO-HNS[1] (hearing class)	
A	1
B	2
C	0
D	0
DS[2]	1
Without preoperative hearing class	1
Mean tumor volume (cm^3) and SD	5.3 ± 6.19 (range: 0.1-15.9)
Median tumor volume (cm^3) and IQR	1.6 (8.77)

[1] AAO-HNS: American Academy of Otolaryngology-Head and Neck Surgery.

Supplemental method 1. Preparation of cryosections.

Once the tumor samples were removed from the patients, they were directly frozen at -150 °C, embedded in Tissue-Tek O.C.T. Compound (Sakura Finetek, Torrance, CA, United States) cooled with dry ice and stored at -80 °C. Tissue sections were prepared using a cryomicrotome (5 µm; CM 3050 S; Leica, Wetzlar, Germany). These were transferred straight to a microscope slide and immersed in acetone (Carl Roth GmbH+Co. KG, Karlsruhe, Germany) for 10 sec, which evaporated at room temperature. The cryosections were stored at -80 °C.

Supplemental method 2. Immunohistochemical staining.

The Dako REAL™ Detection System, Alkaline Phosphatase/RED, Rabbit/Mouse kit (Dako Denmark A/S, Denmark) was used for immunohistochemical staining (IHC) according to the manufacturer's instructions. The cryosections used were prepared as

described in supplemental method 1. Briefly, the cryosections were incubated with the primary antibody (Table S5). Interrupted by washing steps, the sections were incubated with biotinylated secondary antibody and streptavidin alkaline phosphatase. The sections were then stained with red chromogens and finally hematoxylin (Dako Denmark A/S, Denmark).

Table S5. Antibody used for immunohistochemistry.

Antibody	Source	Dilution	Manufacturer
S100B	Mouse	1:200	Novus Biologicals

Table S6. Antibodies used for MELC.

Antibody	Source / Isotype	Dilution	Manufacturer
CD3	Mouse IgG	1:20	ImmunoTools
CD4	Mouse IgG	1:20	ImmunoTools
CD8	Mouse IgG	1:40	ImmunoTools
CD14	Mouse IgG	1:40	ImmunoTools
CD16	Mouse IgG	1:20	Beckman Coulter
CD25	Mouse IgG	1:20	ImmunoTools
CD40	Mouse IgG	1:40	ImmunoTools
CD45	Mouse IgG	1:20	ImmunoTools
CD56	Mouse IgG	1:40	ImmunoTools
CD68	Mouse IgG	1:40	Dako
CD86	Rabbit IgG	1:50	abcam
CD80	Rabbit IgG	1:40	abcam
CD163	Mouse IgG	1:20	BioLegend
CD274	Rat IgG	1:40	BioLegend
CD279	Human IgG	1:40	Miltenyi Biotec
CTLA4	Mouse IgG	1:40	Novus Biologicals

Table S7. Classification of VS developed by Koos.

Tumor grade	Description of tumor extent
Grade 1	Intrameatal tumor
Grade 2	Tumor protrusion towards the cerebellopontine angle without contact with the brain stem
Grade 3	Tumor extends to the brain stem but does not displace it
Grade 4	Tumor displaces brain stem and cranial nerves

Table S8. Mean expression of CD56 in VS samples used for MELC.

Sample	CD56 [% of total cell count] (SD)
Small VS	
1	70.22 (0.24)
2	85.32 (7.88)
3	76.43 (2.73)
Large VS	
4	76.54 (11.43)
5	80.71 (9.83)

Table S9. Spearman's r and p values for correlation analysis of investigated surface markers and clinical parameters in 204 patients with sporadic VS.

Row	Column	Spearman's r	p value

Age at surgery	Koos	0.077	0.278
Age at surgery	Hearing class	0.400	**<0.001**
Koos	Hearing class	0.165	**0.024**
Age at surgery	Tumor volume	0.141	0.060
Koos	Tumor volume	0.896	**<0.001**
Hearing class	Tumor volume	0.191	**0.013**
Age at surgery	*CD247*	0.104	0.218
Koos	*CD247*	0.246	**0.003**
Hearing class	*CD247*	0.114	0.197
Tumor volume	*CD247*	0.285	**0.001**
Age at surgery	*PTPRC*	0.219	**0.024**
Koos	*PTPRC*	0.277	**0.004**
Hearing class	*PTPRC*	0.222	**0.033**
Tumor volume	*PTPRC*	0.216	**0.039**
CD247	*PTPRC*	0.676	**<0.001**
Age at surgery	*CD68*	0.071	0.315
Koos	*CD68*	0.264	**<0.001**
Hearing class	*CD68*	0.200	**0.006**
Tumor volume	*CD68*	0.250	**<0.001**
CD247	*CD68*	0.660	**<0.001**
PTPRC	*CD68*	0.849	**<0.001**
Age at surgery	*CD163*	0.049	0.493
Koos	*CD163*	0.161	**0.024**
Hearing class	*CD163*	0.158	**0.032**
Tumor volume	*CD163*	0.157	**0.038**
CD247	*CD163*	0.756	**<0.001**
PTPRC	*CD163*	0.805	**<0.001**
CD68	*CD163*	0.803	**<0.001**
Age at surgery	*CD3*	0.249	**0.016**
Koos	*CD3*	0.324	**0.002**
Hearing class	*CD3*	0.102	0.373
Tumor volume	*CD3*	0.279	**0.012**
CD247	*CD3*	0.660	**<0.001**
PTPRC	*CD3*	0.806	**<0.001**
CD68	*CD3*	0.770	**<0.001**
CD163	*CD3*	0.749	**<0.001**
Age at surgery	*CD4*	0.160	0.133
Koos	*CD4*	0.351	**<0.001**
Hearing class	*CD4*	0.187	0.104
Tumor volume	*CD4*	0.297	**0.009**
CD247	*CD4*	0.525	**<0.001**
PTPRC	*CD4*	0.662	**<0.001**
CD68	*CD4*	0.677	**<0.001**
CD163	*CD4*	0.604	**<0.001**
CD3	*CD4*	0.697	**<0.001**
Age at surgery	*CD8*	0.097	0.387
Koos	*CD8*	0.193	0.087
Hearing class	*CD8*	0.093	0.453
Tumor volume	*CD8*	0.176	0.143
CD247	*CD8*	0.547	**<0.001**
PTPRC	*CD8*	0.798	**<0.001**
CD68	*CD8*	0.723	**<0.001**
CD163	*CD8*	0.685	**<0.001**

https://doi.org/10.3390/cancers18030355

CD3	*CD8*	0.874	**<0.001**
CD4	*CD8*	0.648	**<0.001**
Age at surgery	*CD14*	0.242	**0.027**
Koos	*CD14*	0.336	**0.002**
Hearing class	*CD14*	0.093	0.442
Tumor volume	*CD14*	0.353	**0.002**
CD247	*CD14*	0.776	**<0.001**
PTPRC	*CD14*	0.776	**<0.001**
CD68	*CD14*	0.789	**<0.001**
CD163	*CD14*	0.793	**<0.001**
CD3	*CD14*	0.758	**<0.001**
CD4	*CD14*	0.620	**<0.001**
CD8	*CD14*	0.656	**<0.001**
Age at surgery	*CD40*	0.240	**0.022**
Koos	*CD40*	0.196	0.064
Hearing class	*CD40*	0.143	0.215
Tumor volume	*CD40*	0.220	0.051
CD247	*CD40*	0.757	**<0.001**
PTPRC	*CD40*	0.571	**<0.001**
CD68	*CD40*	0.554	**<0.001**
CD163	*CD40*	0.719	**<0.001**
CD3	*CD40*	0.522	**<0.001**
CD4	*CD40*	0.568	**<0.001**
CD8	*CD40*	0.439	**<0.001**
CD14	*CD40*	0.755	**<0.001**
Age at surgery	*FCGR3A*	0.224	**0.040**
Koos	*FCGR3A*	0.371	**<0.001**
Hearing class	*FCGR3A*	0.141	0.238
Tumor volume	*FCGR3A*	0.328	**0.005**
CD247	*FCGR3A*	0.742	**<0.001**
PTPRC	*FCGR3A*	0.920	**<0.001**
CD68	*FCGR3A*	0.899	**<0.001**
CD163	*FCGR3A*	0.864	**<0.001**
CD3	*FCGR3A*	0.856	**<0.001**
CD4	*FCGR3A*	0.821	**<0.001**
CD8	*FCGR3A*	0.818	**<0.001**
CD14	*FCGR3A*	0.874	**<0.001**
CD40	*FCGR3A*	0.696	**<0.001**
Age at surgery	*PDCD1*	0.137	0.204
Koos	*PDCD1*	0.221	**0.041**
Hearing class	*PDCD1*	0.105	0.368
Tumor volume	*PDCD1*	0.287	**0.012**
CD247	*PDCD1*	0.589	**<0.001**
PTPRC	*PDCD1*	0.465	**<0.001**
CD68	*PDCD1*	0.400	**<0.001**
CD163	*PDCD1*	0.520	**<0.001**
CD3	*PDCD1*	0.528	**<0.001**
CD4	*PDCD1*	0.370	**<0.001**
CD8	*PDCD1*	0.476	**<0.001**
CD14	*PDCD1*	0.622	**<0.001**
CD40	*PDCD1*	0.692	**<0.001**
FCGR3A	*PDCD1*	0.493	**<0.001**

Table S10. Mann Whitney test of *PTPRC* RNA level in VS tumor samples.

Table Analyzed	*PTPRC* (CD45)
Column B	small VS
vs.	vs.
Column A	large VS
Mann Whitney test	
P value	0.0167
Exact or approximate P value?	Exact
P value summary	*
Significantly different (P < 0.05)?	Yes
One- or two-tailed P value?	Two-tailed
Sum of ranks in column A, B	27, 28
Mann-Whitney U	0
Difference between medians	
Median of column A	8.529, n=3
Median of column B	2.990, n=7
Difference: Actual	-5.539
Difference: Hodges-Lehmann	-5.708

Table S11. Mann Whitney test of CD45 expression detected in MELC images in large and small VS.

Table Analyzed	CD45
Column B	large VS
vs.	vs.
Column A	small VS
Mann Whitney test	
P value	0.011
Exact or approximate P value?	Exact
P value summary	*
Significantly different (P < 0.05)?	Yes
One- or two-tailed P value?	Two-tailed
Sum of ranks in column A, B	62, 74
Mann-Whitney U	7
Difference between medians	
Median of column A	18.98, n=10
Median of column B	33.06, n=6
Difference: Actual	14.08
Difference: Hodges-Lehmann	11.74

Table S12. Mann Whitney test of *CD14* RNA level in VS tumor samples.

Table Analyzed	*CD14*
Column B	small VS
vs.	vs.
Column A	large VS
Mann Whitney test	
P value	0.0667
Exact or approximate P value?	Exact
P value summary	ns
Significantly different (P < 0.05)?	No
One- or two-tailed P value?	Two-tailed
Sum of ranks in column A, B	25, 30
Mann-Whitney U	2
Difference between medians	

Median of column A	92.99, n=3
Median of column B	21.19, n=7
Difference: Actual	-71.8
Difference: Hodges-Lehmann	-53.3

Table S13. Mann Whitney test of *FCGR3A* (CD16) RNA level in VS tumor samples.

Table Analyzed	*FCGR3A* (CD16)
Column B	small VS
vs.	vs.
Column A	large VS
Mann Whitney test	
P value	0.0167
Exact or approximate P value?	Exact
P value summary	*
Significantly different (P < 0.05)?	Yes
One- or two-tailed P value?	Two-tailed
Sum of ranks in column A, B	27, 28
Mann-Whitney U	0
Difference between medians	
Median of column A	68.66, n=3
Median of column B	15.22, n=7
Difference: Actual	-53.44
Difference: Hodges-Lehmann	-56.76

Table S14. Mann Whitney test of *CD68* RNA level in VS tumor samples.

Table Analyzed	*CD68*
Column B	small VS
vs.	vs.
Column A	large VS
Mann Whitney test	
P value	0.0167
Exact or approximate P value?	Exact
P value summary	*
Significantly different (P < 0.05)?	Yes
One- or two-tailed P value?	Two-tailed
Sum of ranks in column A, B	27, 28
Mann-Whitney U	0
Difference between medians	
Median of column A	102.0, n=3
Median of column B	25.27, n=7
Difference: Actual	-76.78
Difference: Hodges-Lehmann	-76.78

Table S15. Mann Whitney test of *CD163* RNA level in VS tumor samples.

Table Analyzed	*CD163*
Column B	small VS
vs.	vs.
Column A	large VS
Mann Whitney test	
P value	0.0667
Exact or approximate P value?	Exact
P value summary	ns
Significantly different (P < 0.05)?	No

https://doi.org/10.3390/cancers18030355

One- or two-tailed P value?	Two-tailed
Sum of ranks in column A, B	25, 30
Mann-Whitney U	2
Difference between medians	
Median of column A	65.87, n=3
Median of column B	22.60, n=7
Difference: Actual	-43.27
Difference: Hodges-Lehmann	-45.7

Table S16. Mann Whitney test of relative total macrophage composition in VS tumor samples.

Table Analyzed	Total macrophages
Column B	large VS
vs.	vs.
Column A	small VS
Mann Whitney test	
P value	0.0167
Exact or approximate P value?	Exact
P value summary	*
Significantly different (P < 0.05)?	Yes
One- or two-tailed P value?	Two-tailed
Sum of ranks in column A, B	28, 27
Mann-Whitney U	0
Difference between medians	
Median of column A	0.5488, n=7
Median of column B	0.7580, n=3
Difference: Actual	0.2092
Difference: Hodges-Lehmann	0.2013

Table S17. Mann Whitney test of relative monocyte composition in VS tumor samples.

Table Analyzed	Monocytes
Column B	large VS
vs.	vs.
Column A	small VS
Mann Whitney test	
P value	0.1167
Exact or approximate P value?	Exact
P value summary	ns
Significantly different (P < 0.05)?	No
One- or two-tailed P value?	Two-tailed
Sum of ranks in column A, B	31, 24
Mann-Whitney U	3
Difference between medians	
Median of column A	0.1288, n=7
Median of column B	0.2112, n=3
Difference: Actual	0.08237
Difference: Hodges-Lehmann	0.08237

Table S18. Mann Whitney test of relative M1 macrophage composition in VS tumor samples.

Table Analyzed	M1 macrophages
Column B	large VS
vs.	vs.
Column A	small VS
Mann Whitney test	

https://doi.org/10.3390/cancers18030355

P value	0.1750
Exact or approximate P value?	Exact
P value summary	ns
Significantly different (P < 0.05)?	No
One- or two-tailed P value?	Two-tailed
Sum of ranks in column A, B	32, 23
Mann-Whitney U	4
Difference between medians	
Median of column A	0.002535, n=7
Median of column B	0.01783, n=3
Difference: Actual	0.01530
Difference: Hodges-Lehmann	0.01530

Table S19. Mann Whitney test of relative M2 macrophage composition in VS tumor samples.

**Table Analyzed**	**M2 macrophages**
Column B	large VS
vs.	vs.
Column A	small VS
Mann Whitney test	
P value	0.1833
Exact or approximate P value?	Exact
P value summary	ns
Significantly different (P < 0.05)?	No
One- or two-tailed P value?	Two-tailed
Sum of ranks in column A, B	32, 23
Mann-Whitney U	4
Difference between medians	
Median of column A	0.4949, n=7
Median of column B	0.5260, n=3
Difference: Actual	0.03109
Difference: Hodges-Lehmann	0.03414

Table S20. Mann Whitney test of CD68 expression detected in MELC images in large and small VS.

**Table Analyzed**	**CD68**
Column B	large VS
vs.	vs.
Column A	small VS
Mann Whitney test	
P value	0.001
Exact or approximate P value?	Exact
P value summary	***
Significantly different (P < 0.05)?	Yes
One- or two-tailed P value?	Two-tailed
Sum of ranks in column A, B	57, 79
Mann-Whitney U	2
Difference between medians	
Median of column A	7.416, n=10
Median of column B	25.97, n=6
Difference: Actual	18.56
Difference: Hodges-Lehmann	17.82

https://doi.org/10.3390/cancers18030355

Table S21. Mann Whitney test of CD40 expression detected in MELC images in large and small VS.

Table Analyzed	CD40
Column B<br>vs.<br>Column A	large VS<br>vs.<br>small VS
Mann Whitney test	
P value	0.007
Exact or approximate P value?	Exact
P value summary	**
Significantly different (P < 0.05)?	Yes
One- or two-tailed P value?	Two-tailed
Sum of ranks in column A, B	48, 57
Mann-Whitney U	3
Difference between medians	
Median of column A	0.6551, n=9
Median of column B	1.516, n=5
Difference: Actual	0.8608
Difference: Hodges-Lehmann	0.84

Table S22. Mann Whitney test of CD163 expression detected in MELC images in large and small VS.

Table Analyzed	CD163
Column B<br>vs.<br>Column A	large VS<br>vs.<br>small VS
Mann Whitney test	
P value	0.0007
Exact or approximate P value?	Exact
P value summary	***
Significantly different (P < 0.05)?	Yes
One- or two-tailed P value?	Two-tailed
Sum of ranks in column A, B	36, 69
Mann-Whitney U	0
Difference between medians	
Median of column A	7.942, n=8
Median of column B	16.95, n=6
Difference: Actual	9.008
Difference: Hodges-Lehmann	9.324

Table S23. Mann Whitney test of $CD40^+CD68^+$ expression detected in MELC images in large and small VS.

Table Analyzed	$CD68^+CD40^+$
Column B<br>vs.<br>Column A	large VS<br>vs.<br>small VS
Mann Whitney test	
P value	0.0126
Exact or approximate P value?	Exact
P value summary	*
Significantly different (P < 0.05)?	Yes
One- or two-tailed P value?	Two-tailed
Sum of ranks in column A, B	63, 73

https://doi.org/10.3390/cancers18030355

Mann-Whitney U	8
Difference between medians	
Median of column A	0.000, n=10
Median of column B	0.06956, n=6
Difference: Actual	0.06956
Difference: Hodges-Lehmann	0.06256

Table S24. Mann Whitney test of CD163+CD68+ expression detected in MELC images in large and small VS.

**Table Analyzed**	**CD68+CD163+**
Column B	large VS
vs.	vs.
Column A	small VS
Mann Whitney test	
P value	0.0302
Exact or approximate P value?	Exact
P value summary	*
Significantly different (P < 0.05)?	Yes
One- or two-tailed P value?	Two-tailed
Sum of ranks in column A, B	65, 71
Mann-Whitney U	10
Difference between medians	
Median of column A	0.4162, n=10
Median of column B	1.535, n=6
Difference: Actual	1.118
Difference: Hodges-Lehmann	0.946

Table S25. Mann Whitney test of CD14+CD68+ expression detected in MELC images in large and small VS.

**Table Analyzed**	**CD68+CD14+**
Column B	large VS
vs.	vs.
Column A	small VS
Mann Whitney test	
P value	0.1179
Exact or approximate P value?	Exact
P value summary	ns
Significantly different (P < 0.05)?	No
One- or two-tailed P value?	Two-tailed
Sum of ranks in column A, B	70, 66
Mann-Whitney U	15
Difference between medians	
Median of column A	0.9387, n=10
Median of column B	1.621, n=6
Difference: Actual	0.682
Difference: Hodges-Lehmann	0.8278

Table S26. Mann Whitney test of CD16+CD68+ expression detected in MELC images in large and small VS.

**Table Analyzed**	**CD68+CD16+**
Column B	large VS
vs.	vs.

https://doi.org/10.3390/cancers18030355

Column A	small VS
Mann Whitney test	
P value	0.2198
Exact or approximate P value?	Exact
P value summary	ns
Significantly different (P < 0.05)?	No
One- or two-tailed P value?	Two-tailed
Sum of ranks in column A, B	73, 63
Mann-Whitney U	18
Difference between medians	
Median of column A	0.3369, n=10
Median of column B	0.6371, n=6
Difference: Actual	0.3002
Difference: Hodges-Lehmann	0.2251

Table S27. Mann Whitney test of *CD3D* RNA level in VS tumor samples.

**Table Analyzed**	***CD3D***
Column B	large VS
vs.	vs.
Column A	small VS
Mann Whitney test	
P value	0.1167
Exact or approximate P value?	Exact
P value summary	ns
Significantly different (P < 0.05)?	No
One- or two-tailed P value?	Two-tailed
Sum of ranks in column A, B	31, 24
Mann-Whitney U	3
Difference between medians	
Median of column A	0.4907, n=7
Median of column B	1.036, n=3
Difference: Actual	0.5448
Difference: Hodges-Lehmann	0.6039

Table S28. Mann Whitney test of *CD3G* RNA level in VS tumor samples.

**Table Analyzed**	***CD3G***
Column B	large VS
vs.	vs.
Column A	small VS
Mann Whitney test	
P value	0.0167
Exact or approximate P value?	Exact
P value summary	*
Significantly different (P < 0.05)?	Yes
One- or two-tailed P value?	Two-tailed
Sum of ranks in column A, B	28, 27
Mann-Whitney U	0
Difference between medians	
Median of column A	0.06234, n=7
Median of column B	0.5038, n=3
Difference: Actual	0.4415
Difference: Hodges-Lehmann	0.4415

https://doi.org/10.3390/cancers18030355

Table S29. Mann Whitney test of *CD4* RNA level in VS tumor samples.

**Table Analyzed**	***CD4***
Column B	small VS
vs.	vs.
Column A	large VS
Mann Whitney test	
P value	0.0167
Exact or approximate P value?	Exact
P value summary	*
Significantly different (P < 0.05)?	Yes
One- or two-tailed P value?	Two-tailed
Sum of ranks in column A, B	27, 28
Mann-Whitney U	0
Difference between medians	
Median of column A	31.22, n=3
Median of column B	8.645, n=7
Difference: Actual	-22.57
Difference: Hodges-Lehmann	-21.63

Table S30. Mann Whitney test of *CD8A* RNA level in VS tumor samples.

**Table Analyzed**	***CD8A***
Column B	small VS
vs.	vs.
Column A	large VS
Mann Whitney test	
P value	0.0167
Exact or approximate P value?	Exact
P value summary	*
Significantly different (P < 0.05)?	Yes
One- or two-tailed P value?	Two-tailed
Sum of ranks in column A, B	27, 28
Mann-Whitney U	0
Difference between medians	
Median of column A	1.747, n=3
Median of column B	0.3721, n=7
Difference: Actual	-1.375
Difference: Hodges-Lehmann	-1.173

Table S31. Mann Whitney test of *IL2RA* (CD25) RNA level in VS tumor samples.

**Table Analyzed**	***IL2RA* (CD25)**
Column B	small VS
vs.	vs.
Column A	large VS
Mann Whitney test	
P value	0.0333
Exact or approximate P value?	Exact
P value summary	*
Significantly different (P < 0.05)?	Yes
One- or two-tailed P value?	Two-tailed
Sum of ranks in column A, B	26, 29
Mann-Whitney U	1
Difference between medians	
Median of column A	1.030, n=3

https://doi.org/10.3390/cancers18030355

Median of column B	0.1876, n=7
Difference: Actual	-0.8424
Difference: Hodges-Lehmann	-0.8713

Table S32. Mann Whitney test of relative CD4+ resting memory T cell composition in VS tumor samples.

Table Analyzed	CD4+ resting memory T cells
Column B	large VS
vs.	vs.
Column A	small VS
Mann Whitney test	
P value	0.1833
Exact or approximate P value?	Exact
P value summary	ns
Significantly different (P < 0.05)?	No
One- or two-tailed P value?	Two-tailed
Sum of ranks in column A, B	45, 10
Mann-Whitney U	4
Difference between medians	
Median of column A	0.1366, n=7
Median of column B	0,.8305, n=3
Difference: Actual	-0.05358
Difference: Hodges-Lehmann	-0.05358

Table S33. Mann Whitney test of relative follicular helper T cell composition in VS tumor samples.

Table Analyzed	Follicular helper T cells
Column B	large VS
vs.	vs.
Column A	small VS
Mann Whitney test	
P value	0.1333
Exact or approximate P value?	Exact
P value summary	ns
Significantly different (P < 0.05)?	No
One- or two-tailed P value?	Two-tailed
Sum of ranks in column A, B	46 ,9
Mann-Whitney U	3
Difference between medians	
Median of column A	0.02177, n=7
Median of column B	0.000, n=3
Difference: Actual	-0.02177
Difference: Hodges-Lehmann	-0.02177

Table S34. Mann Whitney test of relative CD4+ active memory T cell composition in VS tumor samples.

Table Analyzed	CD4+ active memory T cells
Column B	large VS
vs.	vs.
Column A	small VS
Mann Whitney test	
P value	0.0667
Exact or approximate P value?	Exact
P value summary	ns

https://doi.org/10.3390/cancers18030355

Significantly different (P < 0.05)?	No
One- or two-tailed P value?	Two-tailed
Sum of ranks in column A, B	31.50, 23.50
Mann-Whitney U	3,500
Difference between medians	
Median of column A	0.000, n=7
Median of column B	0.0008502, n=3
Difference: Actual	0.0008502
Difference: Hodges-Lehmann	0.0008502

Table S35. Mann Whitney test of CD3 expression detected in MELC images in large and small VS.

**Table Analyzed**	**CD3**
Column B	large VS
vs.	vs.
Column A	small VS
Mann Whitney test	
P value	0.0007
Exact or approximate P value?	Exact
P value summary	***
Significantly different (P < 0.05)?	Yes
One- or two-tailed P value?	Two-tailed
Sum of ranks in column A, B	55, 65
Mann-Whitney U	0
Difference between medians	
Median of column A	0.8624, n=10
Median of column B	2.783, n=5
Difference: Actual	1.92
Difference: Hodges-Lehmann	1.956

Table S36. Mann Whitney test of $CD3^+CD4^+$ expression detected in MELC images in large and small VS.

**Table Analyzed**	**$CD3^+CD4^+$**
Column B	large VS
vs.	vs.
Column A	small VS
Mann Whitney test	
P value	0.0027
Exact or approximate P value?	Exact
P value summary	**
Significantly different (P < 0.05)?	Yes
One- or two-tailed P value?	Two-tailed
Sum of ranks in column A, B	57, 63
Mann-Whitney U	2
Difference between medians	
Median of column A	0.09444, n=10
Median of column B	0.5137, n=5
Difference: Actual	0.4193
Difference: Hodges-Lehmann	0.4451

Table S37. Mann Whitney test of CD8 expression detected in MELC images in large and small VS.

**Table Analyzed**	**CD8**
Column B	large VS
vs.	vs.

Column A	small VS
Mann Whitney test	
P value	0.0075
Exact or approximate P value?	Exact
P value summary	**
Significantly different (P < 0.05)?	Yes
One- or two-tailed P value?	Two-tailed
Sum of ranks in column A, B	61, 75
Mann-Whitney U	6
Difference between medians	
Median of column A	0.8554, n=10
Median of column B	2.611, n=6
Difference: Actual	1.756
Difference: Hodges-Lehmann	1.736

Table S38. Mann Whitney test of $CD3^{+}CD8^{+}$ expression detected in MELC images in large and small VS.

**Table Analyzed**	**$CD3^{+}CD8^{+}$**
Column B	large VS
vs.	vs.
Column A	small VS
Mann Whitney test	
P value	0.0007
Exact or approximate P value?	Exact
P value summary	***
Significantly different (P < 0.05)?	Yes
One- or two-tailed P value?	Two-tailed
Sum of ranks in column A, B	55, 65
Mann-Whitney U	0
Difference between medians	
Median of column A	0.5346, n=10
Median of column B	1.814, n=5
Difference: Actual	1.28
Difference: Hodges-Lehmann	1.399

Table S39. Mann Whitney test of CD25 expression detected in MELC images in large and small VS.

**Table Analyzed**	**CD25**
Column B	large VS
vs.	vs.
Column A	small VS
Mann Whitney test	
P value	0.0312
Exact or approximate P value?	Exact
P value summary	*
Significantly different (P < 0.05)?	Yes
One- or two-tailed P value?	Two-tailed
Sum of ranks in column A, B	65, 71
Mann-Whitney U	10
Difference between medians	
Median of column A	0.2807, n=10
Median of column B	1.232, n=6
Difference: Actual	0.9508

Difference: Hodges-Lehmann	0.7438

Table S40. Mann Whitney test of $CD3^{+}CD25^{+}$ expression detected in MELC images in large and small VS.

**Table Analyzed**	**$CD3^{+}CD25^{+}$**
Column B	large VS
vs.	vs.
Column A	small VS
Mann Whitney test	
P value	0.0003
Exact or approximate P value?	Exact
P value summary	***
Significantly different (P < 0.05)?	Yes
One- or two-tailed P value?	Two-tailed
Sum of ranks in column A, B	55, 65
Mann-Whitney U	0
Difference between medians	
Median of column A	0.000, n=10
Median of column B	0.06256, n=5
Difference: Actual	0.06256
Difference: Hodges-Lehmann	0.06256

Table S41. Mann Whitney test of the proportion of $CD274^{+}$ T_c cells in the total T_c cell count detected in MELC images in large and small VS.

**Table Analyzed**	**$CD274^{+}$ T_c cells**
Column B	small VS
vs.	vs.
Column A	large VS
Mann Whitney test	
P value	>0.9999
Exact or approximate P value?	Exact
P value summary	ns
Significantly different (P < 0.05)?	No
One- or two-tailed P value?	Two-tailed
Sum of ranks in column A, B	68, 37
Mann-Whitney U	22
Difference between medians	
Median of column A	39.13, n=9
Median of column B	48.57, n=5
Difference: Actual	9.441
Difference: Hodges-Lehmann	-0.7026

Table S42. Mann Whitney test of $CD56^{+}CD274^{+}CD16^{-}$ expression detected in MELC images in large and small VS.

**Table Analyzed**	**$CD56^{+}CD274^{+}CD16^{-}$**
Column B	large VS
vs.	vs.
Column A	small VS
Mann Whitney test	
P value	0.405
Exact or approximate P value?	Exact
P value summary	*

https://doi.org/10.3390/cancers18030355

Significantly different (P < 0.05)?	Yes
One- or two-tailed P value?	Two-tailed
Sum of ranks in column A, B	66, 70
Mann-Whitney U	11
Difference between medians	
Median of column A	0.2402, n=10
Median of column B	1.878, n=6
Difference: Actual	1.638
Difference: Hodges-Lehmann	1.389

Table S43. Mann Whitney test of CD279 expression detected in MELC images in large and small VS.

Table Analyzed	CD279
Column B	large VS
vs.	vs.
Column A	small VS
Mann Whitney test	
P value	0.012
Exact or approximate P value?	Exact
P value summary	*
Significantly different (P < 0.05)?	Yes
One- or two-tailed P value?	Two-tailed
Sum of ranks in column A, B	51, 69
Mann-Whitney U	6
Difference between medians	
Median of column A	0.3032, n=9
Median of column B	2.525, n=6
Difference: Actual	2.222
Difference: Hodges-Lehmann	1.953

Table S44. Mann Whitney test of $CD3^{+}CD8^{+}CD279^{+}$ expression detected in MELC images in large and small VS.

Table Analyzed	$CD3^{+}CD8^{+}CD279^{+}$
Column B	large VS
vs.	vs.
Column A	small VS
Mann Whitney test	
P value	0.001
Exact or approximate P value?	Exact
P value summary	***
Significantly different (P < 0.05)?	Yes
One- or two-tailed P value?	Two-tailed
Sum of ranks in column A, B	45, 60
Mann-Whitney U	0
Difference between medians	
Median of column A	0.2087, n=9
Median of column B	0.8167, n=5
Difference: Actual	0.6081
Difference: Hodges-Lehmann	0.6178

Table S45. Mann Whitney test of $CTLA4^{+}$ cells detected in MELC images in large and small VS.

Table Analyzed	CTLA4
Column B	large VS

vs.	vs.
Column A	small VS
Mann Whitney test	
P value	0.0295
Exact or approximate P value?	Exact
P value summary	*
Significantly different (P < 0.05)?	Yes
One- or two-tailed P value?	Two-tailed
Sum of ranks in column A, B	41, 50
Mann-Whitney U	5
Difference between medians	
Median of column A	0.1865, n=8
Median of column B	3.573, n=5
Difference: Actual	3.387
Difference: Hodges-Lehmann	3.115

Table S46. Mann Whitney test of *CTLA4* (CD152) RNA level in VS tumor samples.

**Table Analyzed**	***CTLA4* (CD152)**
Column B	small VS
vs.	vs.
Column A	large VS
Mann Whitney test	
P value	0.0167
Exact or approximate P value?	Exact
P value summary	*
Significantly different (P < 0.05)?	Yes
One- or two-tailed P value?	Two-tailed
Sum of ranks in column A, B	27, 28
Mann-Whitney U	0
Difference between medians	
Median of column A	0.1873, n=3
Median of column B	0.04427, n=7
Difference: Actual	-0.143
Difference: Hodges-Lehmann	-0.1554

Disclaimer/Publisher's Note: The statements, opinions and data contained in all publications are solely those of the individual author(s) and contributor(s) and not of MDPI and/or the editor(s). MDPI and/or the editor(s) disclaim responsibility for any injury to people or property resulting from any ideas, methods, instructions or products referred to in the content.
